# Mechanistic insights into the diuretic activity of sugarcane leaf extract using untargeted metabolomics and bioinformatics

**DOI:** 10.3389/fpls.2025.1652526

**Published:** 2025-09-08

**Authors:** Xianglong Pan, Erwei Hao, Jinling Xie, Pei Ling Tang, Juan Wen, Zhengcai Du, Jiagang Deng, Xiaotao Hou

**Affiliations:** ^1^ Department of Pharmaceutical, Heilongjiang University of Chinese Medicine, Harbin, China; ^2^ Guangxi Key Laboratory of Efficacy Study on Chinese Material Medical, Guangxi University of Chinese Medicine, Nanning, China; ^3^ University Engineering Research Center of Reutilization of Traditional Chinese Medicine Resources, Guangxi University of Chinese Medicine, Nanning, Guangxi, China; ^4^ Guangxi Key Laboratory of Traditional Chinese Medicine (TCM) Formulas Theory and Transformation for Damp Diseases, Guangxi University of Chinese Medicine, Nanning, China; ^5^ Department of Bioscience, Faculty of Applied Sciences, Tunku Abdul Rahman University of Management and Technology, Kuala Lumpur, Malaysia

**Keywords:** sugarcane leaf, diuretic activity, untargeted metabolomics, network pharmacology, molecular dynamics simulations, molecular docking

## Abstract

**Introduction:**

Sugarcane leaf (*Poaceae*) is a widely cultivated herbaceous plant in the tropical regions of southern China. Traditional Yao medicine has utilized its significant diuretic effects for the treatment of edema-related diseases. However, the underlying mechanisms of its diuretic activity remain unclear.

**Methods:**

This study aims to elucidate the potential mechanisms of the diuretic activity of sugarcane leaf extract using untargeted metabolomics, network pharmacology, and molecular dynamics simulations.

**Results and discussion:**

A water-loaded rat model was established to assess diuresis, and sugarcane leaf extract markedly increased urinary excretion of Na^+^, Cl^-^, and other ions. Ultra-performance liquid chromatography–mass spectrometry (UPLC–MS) identified ten absorbed constituents in rat serum after sugarcane leaf extract administration. Untargeted metabolomics revealed ten endogenous differential metabolites and two key metabolic pathways modulated by sugarcane leaf extract. Network pharmacology uncovered 63 overlapping targets, among which AKT1, IL-6, TNF, STAT3, and EGFR were pinpointed as core targets implicated in the diuretic response. Molecular docking and dynamics simulations confirmed strong binding affinities between these core targets and five absorbed sugarcane leaf extract constituents. The alanine, aspartate, and glutamate metabolism pathway was highlighted as pivotal for sugarcane leaf extract-induced diuresis. Collectively, this study has elucidated the diuretic mechanism of sugarcane leaf extract, providing a scientific basis for its clinical application and the development of novel diuretic agents.

## 1 Introduction

Diuretics are a class of drugs widely used in clinical practice and play a crucial role in regulating water and salt metabolism, maintaining homeostasis, and treating various diseases associated with fluid retention (Sarafidis et al., 2010). Studies have shown that their mechanism of action involves promoting sodium and water excretion by the kidneys. This helps regulate electrolyte balance, lower blood pressure, and alleviate edema. They are key in treating diseases such as edema-related disorders, heart failure, nephrotic syndrome, and hypertension (Novak and Ellison, 2022; Arumugham and Shahin, 2023; Wile, 2012). In edema, fluid abnormally accumulates in interstitial tissues. This can result from impaired venous or lymphatic return and endocrine disorders (Koirala et al., 2023; Hinrichs and Jensen, 2024). Patients with heart failure often develop fluid retention due to reduced cardiac output, leading to edema and dyspnea (Trullàs et al., 2024, 2019). Diuretics, as a cornerstone of heart failure treatment, can help remove excess fluid, reduce cardiac load, and improve cardiac function (Mullens et al., 2019). Patients with nephrotic syndrome often experience severe edema due to hypoproteinemia caused by massive proteinuria (Frăţilă et al., 2024; Schork et al., 2024). Traditional diuretics work by inhibiting the activity of prostaglandin-degrading enzymes, thereby increasing the levels of prostaglandin E2. This dilates renal blood vessels, reduces vascular resistance, and alleviates interstitial edema, protecting renal tubules from injury (Bell and Mandalia, 2022; Chen et al., 2020). In hypertension management, diuretics remain cornerstone antihypertensive agents. They reduce blood volume and cardiac output through natriuresis, resulting in lowered blood pressure. Among them, thiazide diuretics are the most widely used and have been shown to significantly reduce the incidence of cardiovascular events and overall mortality (Blowey, 2016). However, long-term use of thiazide diuretics may cause adverse reactions such as hypokalemia, hyperuricemia, and hyperglycemia, increasing the risk of metabolic disorders in patients. Currently, a wide variety of diuretics is used in clinical practice. Based on their mechanisms and sites of action, they are primarily categorized as thiazide diuretics, loop diuretics, potassium-sparing diuretics, carbonic anhydrase inhibitors, and osmotic diuretics (Ellison, 2019; Kehrenberg and Bachmann, 2022; Carvalho et al., 2023). Although traditional diuretics have achieved significant therapeutic success, their adverse effects and limitations have prompted researchers to explore safer and more effective alternatives.

Untargeted metabolomics is a cutting-edge technology capable of comprehensively and unbiased detection and analyzing all small-molecule metabolites in a biological system. It does not require predefined target metabolites and can broadly capture metabolite changes under specific physiological or pathological conditions. This provides a powerful tool for screening endogenous differential metabolic biomarkers related to pharmacological effects (Jiménez-Salcedo et al., 2025; Guo et al., 2024). Network pharmacology relies on high-throughput omics data and public database resources, integrating bioinformatics methods such as molecular docking, pathway enrichment analysis, and topological parameter calculation to systematically analyze the dynamic relationships between multi-component traditional Chinese medicine (TCM) and multi-target biological systems. By constructing weighted network models, it not only visually demonstrates the interaction strength between active components and potential targets but also identifies key functional clusters through modular analysis, thereby revealing the synergistic regulatory mechanism of “multi-component, multi-target, multi-pathway” in TCM’s therapeutic effects on diseases (Zhao et al., 2023). Molecular dynamics simulation is a computational method based on classical mechanics that predicts molecular system particle trajectories and interactions by numerically solving Newton’s equations of motion. It has become essential for analyzing biomacromolecule dynamics and drug-target binding modes (Zheng et al., 2025). In summary, integrating untargeted metabolomics, network pharmacology, and molecular dynamics simulation provides novel approaches to systematically elucidate TCM’s holistic pharmacological characteristics and mechanisms of action.

Sugarcane leaf (*Saccharum sinense L*, Poaceae) is the dried leaf of this grass family plant. In TCM theory, it has the effects of clearing heat, promoting fluid production, exerting natriuretic effects, and relieving dampness-dominant arthralgia. It is commonly used to treat diabetes (consumptive thirst), night sweats, urolithiasis, pruritus, and dental caries (Lu et al., 2025; Liang et al., 2024). Sugarcane leaves contain various bioactive compounds including flavonoids, organic acids, lignans, terpenoids, phenolics, and polysaccharides (Tang et al., 2022; David and Krishnamurthi, 2024). Modern pharmacological studies demonstrate that sugarcane leaves exhibit multiple biological activities including hypoglycemic, anti-inflammatory, antimicrobial, antioxidant, antitumor, and cardioprotective effects (Sun et al., 2023; Tang et al., 2019; Mo et al., 2024). Recent studies on natural plant resources have increasingly focused on sugarcane leaf pharmacology. Studies indicate that sugarcane leaf aqueous extract exhibits hypoglycemic, antimicrobial, and anti-inflammatory effects, suggesting promising medical applications (Hao et al., 2018). Notably, sugarcane leaves have demonstrated significant diuretic potential. Compared to conventional diuretics, sugarcane leaves offer advantages including abundant availability, lower toxicity, fewer side effects, and multi-component synergy (Mo et al., 2023). Unlike traditional diuretics that target single pathways, sugarcane leaves may regulate water-electrolyte balance and diuresis through multi-target synergism. This multi-target approach may enhance efficacy while minimizing adverse effects. However, the diuretic mechanism of sugarcane leaves remains unclear, limiting their clinical translation. This study therefore employs a water-loading rat model combined with serum pharmacochemistry, untargeted metabolomics, network pharmacology, and molecular dynamics simulations to elucidate sugarcane leaves’ diuretic mechanisms at metabolomic and molecular levels. This research will advance understanding of sugarcane leaves’ medicinal value, facilitate their pharmaceutical development, and inform the discovery of safer natural diuretics.

## 2 Materials and methods

### 2.1 Reagent

Mass spectrometry-grade methanol, acetonitrile, and formic acid were purchased from Thermo Fisher Scientific (USA). Sodium ion (Product No. C002-1-1), chloride ion (Product No. C003-2-1), potassium ion (Product No. C001-2-1), creatinine (CRE, Product No. C011-2-1), and blood urea nitrogen (BUN, Product No. C013-2-1) were purchased from Nanjing Jiancheng Bioengineering Institute. Antidiuretic hormone (ADH, Product No. E-EL-R0522), atrial natriuretic peptide (ANP, Product No. E-EL-R0017), renin (REN, Product No. E-EL-R0030), angiotensin II (Ang II, Product No. E-EL-R1430), aldosterone (ALD, Product No. E-EL-0070), cyclic adenosine monophosphate (cAMP, Product No. E-EL-0056), and cyclic guanosine monophosphate (cGMP, Product No. E-EL-0083) were purchased from Wuhan Eliruit Biotechnology Co., Ltd. Hydrochlorothiazide (Product No. 2212020) were purchased from Tianjin Lisheng Pharmaceutical Co., Ltd.

### 2.2 Preparation of plant extracts

Fresh sugarcane leaves were collected from Liucheng County, Liuzhou City, Guangxi Zhuang Autonomous Region, China. The sugarcane leaves were processed at the Guangxi Key Laboratory of Efficacy Study on Chinese Materia Medica to obtain sugarcane leaf extract. The dried sugarcane leaves were cut into small pieces and coarsely ground. An 8–10-fold volume of purified water was added and cold-soaked for 2 h. The decoction was extracted three times at 100°C for 2 h each. The filtrates were combined, concentrated to a specific gravity of 1.15, and stored at 4°C as sugarcane leaf extract for later use.

### 2.3 Screening of experimental animal models

Forty male Sprague-Dawley (SD) rats (SPF-grade, 6 weeks old, and weighing 200 ± 20 g) were purchased from Hunan Slake Jingda Laboratory Animals Co., Ltd. (Animal Production License No. SCXK (Xiang) 2021-0002). Animals were housed in the Experimental Animal Center of Guangxi University of Chinese Medicine. After a 3-day acclimatization period, rats were fasted (with free access to water) for 18 h. Gentle abdominal pressure was applied to expel residual urine from the bladder. Rats received 2.5 mL/100 g body weight of 0.9% sodium chloride solution by oral gavage. Cumulative 2 h urine output was collected. Rats exhibiting a urine volume exceeding 40% of the administered 0.9% sodium chloride solution volume were considered qualified for the experimental animal model.

### 2.4 Animal grouping and administration

Thirty-six qualified rats selected through screening were randomly divided into six groups (n=6) based on body weight: blank control (Con), model (Mod), positive control (Pos), high-dose sugarcane leaf extract (Hig), middle-dose sugarcane leaf extract (Mid), and low-dose sugarcane leaf extract (Low). The Mod group received 0.9% NaCl (20 mL/kg), while other groups were given corresponding treatments (20 mL/kg) once daily for 7 days. Dosage settings: The Pos (hydrochlorothiazide) and the Low were set at clinically equivalent doses (0.0214 g/kg and 3.15 g/kg, respectively). The Mid was 3 times the Low (9.45 g/kg), and the Hig was 9 times the Low (28.35 g/kg) sugarcane leaf extract.

### 2.5 Sample collection and detection

Prior to the final drug administration, all animals were fasted for 12 h with ad libitum water access. Following suprapubic compression to empty bladder residual urine, rats received 25 mL/kg 0.9% NaCl via oral gavage to establish the water-loading model. After 30 min, the model group and treatment groups received their respective drug solutions via oral gavage and were then transferred to metabolic cages. Urine was collected at 1, 2, 4, 6, 12, and 24 h for the detection of urinary indicators. After 24 h, rats were anesthetized by intraperitoneal injection of 10% chloral hydrate. Blood was collected via abdominal aorta, allowed to clot for 1 hour at room temperature, and then centrifuged at 13,000 rpm for 15 min to isolate serum. Throughout the treatment period, daily observations included mental state, food intake, spontaneous activity, and coat condition. Body weight, water intake, and urine output were measured on days 0, 1, 3, 5, and 7 before treatment.

### 2.6 Preparation of urine and serum samples

On the final experimental day, urine samples from all groups were collected, centrifuged at 13,000 rpm for 15 min at 4°C, and the supernatants stored at -80°C. Prior to analysis, samples were thawed to room temperature. A volume of 200 μL of urine sample was transferred to a 1.5 mL centrifuge tube, followed by the addition of 800 μL of ultrapure water. The mixture was vortexed for 1 minute and centrifuged at 13,000 rpm for 15 min. The supernatant was filtered (0.22 μm) for UPLC–MS analysis. Pooled serum was from the Hig group, and 1 mL was transferred into 5 mL EP tubes. Three times the volume of methanol-acetonitrile (2:1, v/v) was added to precipitate proteins. The mixture was vortexed for 3 min and then centrifuged at 13,000 rpm for 8 min at 4°C. The supernatant was filtered through a 0.22 μm microporous membrane and concentrated to dryness using a vacuum freeze-dryer. The residue was reconstituted in 200 μL of 50% methanol by ultrasonication for 30 min, then centrifuged at 13,000 rpm for 15 min at 4°C. The cleared supernatant was analyzed by UPLC–MS. Serum samples from the blank group were processed using the same protocol.

### 2.7 UPLC–MS chromatographic and mass spectrometric conditions

Ultra-high-performance liquid chromatography was performed using an ExionLC AC system (SCIEX, USA) coupled with a QTOF mass spectrometer (X500R; SCIEX, USA) equipped with a Waters ACQUITY UPLC HSS T3 C18 column (1.8 μm 2.1×100 mm).

For serum analysis, the chromatographic conditions were as follows: the mobile phase consisted of 0.1% formic acid in water (A) and 0.1% formic acid in acetonitrile (B). The gradient elution program was: 0–30 min, 5%–95% B; 30–32 min, 95% B; 32–33 min, 95%–5% B; and 33–35 min, 5% B. The column temperature was maintained at 40°C, the injection chamber at 4°C, with a flow rate of 0.4 mL/min and an injection volume of 3 μL. Mass spectrometry was conducted using an electrospray ionization (ESI) source in both positive and negative ion switching modes. The TOF MS scan range was set from *m/z* 100 to 1500. Source parameters included a voltage of –4500 V for negative ions and 5500 V for positive ions, a source temperature of 600°C, GS1/GS2 at 55 psi, and curtain gas at 35 psi. Collision energy was ± 10 V for precursor ions, and ± 35 V for product ions, with a declustering potential (DP) of ± 80 V.

For the untargeted metabolomics section, the mobile phases were identical: 0.1% formic acid in water (A) and 0.1% formic acid in acetonitrile (B). The gradient elution program was as follows: 0–2.5 min, 1%–10% B; 2.5–5.0 min, 10%–20% B; 5.0–7.0 min, 20%–40% B; 7.0–9.5 min, 40%–99% B; 9.5–12.0 min, 99% B; 12.0–13.0 min, 99%–1% B; and 13.0–14.0 min, 1% B. The column temperature was maintained at 40°C, the injection chamber temperature at 4°C, with a flow rate of 0.4 mL/min, and injection volume of 4 μL. MS conditions were identical to those used in the serum analysis.

### 2.8 Histopathological analysis of kidney tissue

Following completion of drug treatment, the rats were anesthetized with 10% chloral hydrate. Bilateral kidneys were excised, weighed, and perfused with ice-cold saline to remove blood residues. The tissues were then fixed in 4% paraformaldehyde solution for 24 h. After fixation, tissues were processed through graded ethanol dehydration, embedded in paraffin, and sectioned. Sections were stained with hematoxylin and eosin (H&E) for histology evaluation and with Masson’s trichrome for fibrosis assessment. Renal cortical morphology was examined under a light microscope.

### 2.9 Untargeted metabolomics analysis

To characterize endogenous metabolic alterations in model rats after sugarcane leaf extract administration, untargeted metabolomics was performed. Urinary metabolite profiles before and after treatment were compared to identify differential biomarkers and associated metabolic pathways. UPLC–MS raw data were processed for peak alignment, retention time correction, and peak area extraction. The resulting data were subjected to principal component analysis (PCA) and orthogonal partial least squares-discriminant analysis (OPLS-DA). Metabolites with variable importance in projection (VIP) scores >1 were preliminarily selected. Significant metabolites were identified by Student’s t-test (*P* < 0.05). Metabolite identification was performed using the Human Metabolome Database (HMDB) and the Kyoto Encyclopedia of Genes and Genomes (KEGG) database. Pathway enrichment analysis was conducted using MetaboAnalyst 5.0.

### 2.10 Network pharmacology analysis

Potential targets of sugarcane leaf absorbed components were identified using Swiss Target Prediction (http://www.swisstargetprediction.ch/) and SEA (Similarity Ensemble Approach) (https://sea.bkslab.org/) databases, with species restricted to “*Homo sapiens*”. Disease-related targets were retrieved from GeneCards (https://www.genecards.org/), OMIM (https://www.omim.org/), and DisGeNET (https://www.disgenet.org/search) using the search keywords: “Edema, Ed”, “Hypertension, Hy”, “Heart failure, Hf” and “Nephrotic syndrome, Ns”. Duplicate targets were removed and overlapping targets between compounds and diseases were identified using Venny 2.1.0 (http://www.liuxiaoyuyuan.cn/). These common targets were considered potential mediators of the diuretic effects of sugarcane leaf extract. Venn diagrams were generated to visualize the overlap between compound and disease targets.

To further explore the interaction among these targets, a protein-protein interaction (PPI) network was constructed using STRING (https://cn.string-db.org/cgi/input). The topological properties of each node in the PPI network were analyzed using the Centiscape 2.2 plugin tool in Cytoscape 3.10.0. Top 15 hub targets were selected based on degree centrality (DC), betweenness centrality (BC), and closeness centrality (CC), identifying key targets potentially involved in the diuretic activity. Gene Ontology (GO) and KEGG enrichment analyses were performed using Metascape (https://metascape.org/gp/index.html), with significance thresholds set at *P <*0.01, minimum count=3 and enrichment factor >1.5. Pathway-associated genes were verified using KEGG database (https://www.kegg.jp/kegg/pathway.html). GO functional annotation and KEGG pathway enrichment results were visualized using the Microbial Information Online platform (http://www.bioinformatics.com.cn). Finally, metabolomics biomarkers and network pharmacology targets were integrated using Integrated Molecular Pathway Level Analysis (IMPaLA, http://impala.molgen.mpg.de/) and visualized in Cytoscape 3.10.0 to construct a comprehensive “biomarkers–components–targets–pathways” network.

### 2.11 Molecular docking

The blood-borne components extracted from sugarcane leaf were selected as small-molecule ligands, while the top five key targets, ranked by Degree in the PPI (Protein-Protein Interaction) network, were chosen as protein receptors for molecular docking, respectively. First, the 2D structures of the blood-borne components were downloaded from the PubChem database (https://pubchem.ncbi.nlm.nih.gov/) and converted into 3D structures using Chem3D software. The optimized small-molecule ligands were saved in MOL2 format. Gene names corresponding to key targets were retrieved from the UniProt database (https://www.uniprot.org/), with the species restricted to *Homo sapiens*. The UniProt IDs were used to search the RCSB PDB database (https://www.rcsb.org/) to acquire the 3D structures of the target proteins, which were saved in PDB format. Protein structures were preprocessed in PyMol software by removing water molecules and adding hydrogen atoms to prepare them for docking. Molecular docking was carried out using AutoDockTools software. Binding affinity was evaluated based on docking energy, with lower binding energy values indicating stronger interactions between the ligands and protein receptors.

### 2.12 Molecular dynamics simulation

Molecular dynamics (MD) simulations were conducted using Gromacs 2022. Small molecules were parameterized with the General Amber Force Field (GAFF), while the protein used the AMBER14SB force field with the TIP3P water model. Systems were prepared by merging protein and ligand structures into complexes. Simulations were conducted under constant temperature and pressure conditions with periodic boundary conditions applied. Bonds involving hydrogen were constrained via LINCS algorithm (2 fs time step). Electrostatic were treated with Particle Mesh Ewald (PME; 1.2 nm cutoff). Van der Waals interactions used 10 Å cutoff (updated every 10 steps). Temperature (298 K) was maintained with v-rescale thermostat, and pressure (1 bar) with Berendsen barostat. The system was first equilibrated with 100 ps of NVT (constant number of particles, volume, and temperature) and NPT (constant number of particles, pressure, and temperature) simulations at 298 K. Subsequently, a 100 ns MD simulation was performed on the complex system, with conformations saved every 10 ps. After the simulation was completed, the trajectories were analyzed using VMD and PyMOL. The binding free energy between the protein and the small-molecule ligand was analyzed using the gmmpbsa program with the Molecular Mechanics Poisson-Boltzmann Surface Area (MMPBSA) method.

To conduct an in-depth analysis of the structural dynamic properties of the protein-ligand complex, root mean square deviation (RMSD), root mean square fluctuation (RMSF), radius of gyration (Rg), and buried solvent accessible surface area (Buried SASA) curves were calculated and plotted. RMSD is used to measure the degree of deviation of the structure from the initial reference structure during the simulation. For the protein, ligand, and protein-ligand complex, the initial conformations of the simulation were used as reference structures, and the RMSD values at each time point were calculated using the gmx rms tool in the Gromacs program. By statistically analyzing and plotting the RMSD values at different time points, the stability of the structure and the trend of conformational changes during the simulation can be visually observed. RMSF reflects the average fluctuation magnitude of each residue in the protein or ligand during the simulation. The RMSF values for each residue were calculated using the gmx rmsf tool, and by plotting the RMSF curves, regions of high flexibility and relative rigidity in the protein, as well as atoms or groups with significant fluctuations in the ligand, can be identified. Rg is an important parameter describing the size and compactness of a molecule. The Rg values for the protein, ligand, and protein-ligand complex at each time point during the simulation were calculated using the gmx gyrate tool, and the Rg curves were plotted. Changes in the Rg curve can reflect variations in the conformational compactness of the molecule during the simulation. SASA represents the area of the molecular surface in contact with solvent molecules, reflecting the solvation properties of the molecule. The SASA values for the protein, ligand, and protein-ligand complex were calculated using the gmx sasa tool, and the SASA curves were plotted. Changes in the SASA curve can reveal dynamic variations in the interactions between the molecule and the solvent during the simulation.

### 2.13 Statistical methods

Data are presented as mean ± standard deviation (x ± s). Statistical analyses were performed using GraphPad Prism 8.4.3. intergroup comparisons used the Student’s t-test. Statistical significance was set at *P* ^#^ < 0.05 and *P* ^##^ < 0.01.

## 3 Results

### 3.1 Identification of phytochemical constituents in sugarcane leaf extract

**Figure 1:**
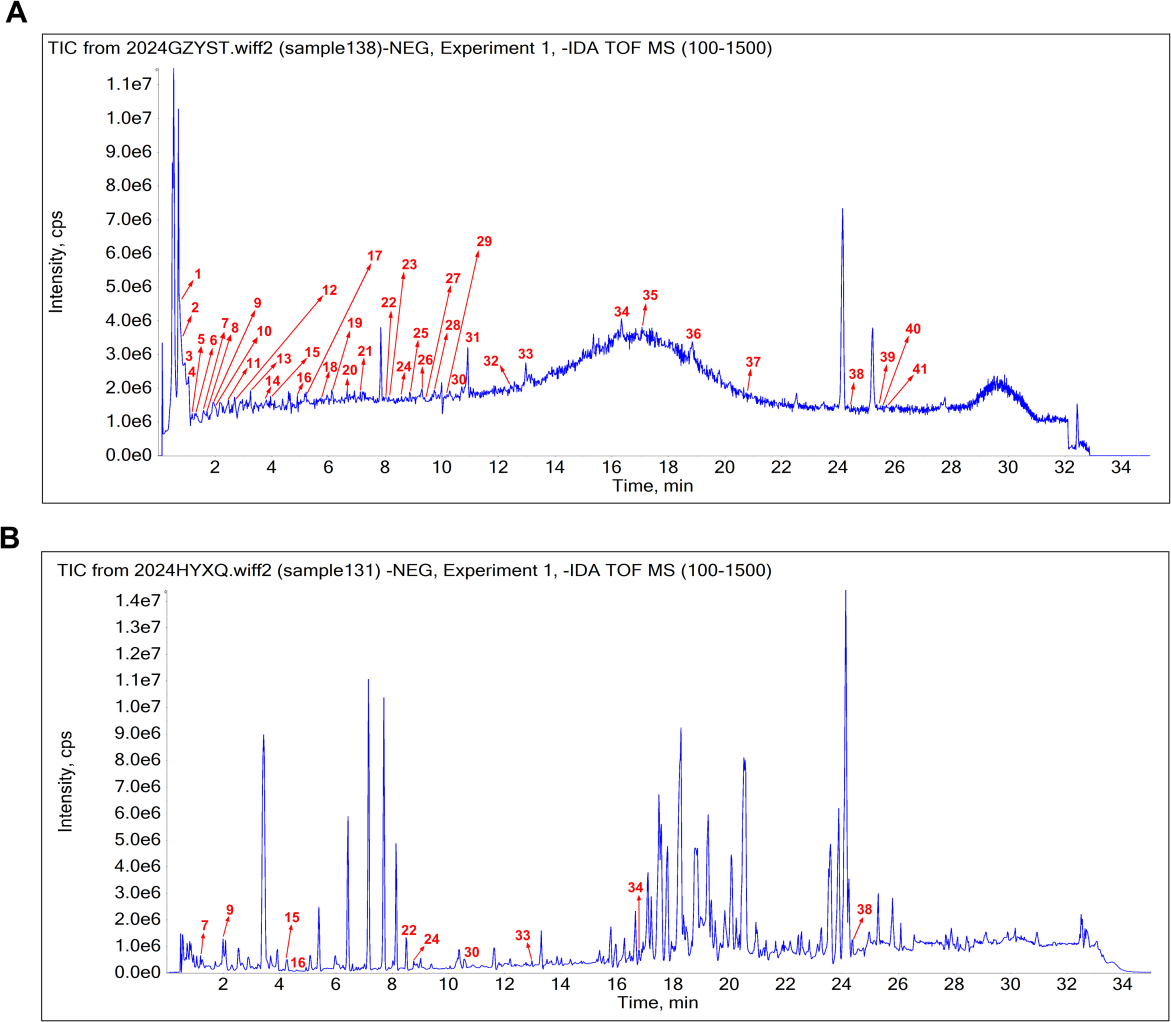
The base peak chromatogram of sugarcane leaf extract. **(A)** Phytochemical constituents identified in sugarcane leaf extract under negative ion mode. **(B)** Absorbed prototype constituents identified in serum.

Phytochemical constituents in sugarcane leaf extract were analyzed by UPLC–MS. The total ion current chromatogram of sugarcane leaf extract is shown in **Figure 1A**. Of 41 phytochemical constituents identified in the negative ion mode, 10 prototype chemical constituents were detected in rat serum (**Figure 1B**). Compounds were characterized through MS/MS spectral interpretation and literature comparison. Identified compounds comprised flavonoids, organic acids, phenols, terpenoids, and saccharides (**Table 1**).

**Table 1:** Constituents identified from sugarcane leaf extract by UPLC–Q–TOF–MS/MS.

No.	Retention time (Rt)	Formula	Adduct/charge	Precursor mass	Found at mass	Mass error (ppm)	Compound name	SDRS	PIS	Fragment ions (*m/z*)
1	0.53	C_6_H_14_O_6_	[M—H]^–^	181.0729	181.0718	0.3	D-Sorbitol	▴	✓	181.0718; 163.0635
2	0.55	C_6_H_12_O_6_	[M—H]^–^	179.0566	179.0561	0.1	D(+)-Glucose	▴	✓	179.0560; 161.0464
3	0.61	C_4_H_6_O_5_	[M—H]^–^	133.0154	133.0146	2.7	Malic acid	▴	✓	133.0147
4	0.68	C_7_H_6_O_5_	[M—H]^–^	169.0156	169.0147	2.8	Gallic acid	▴	✓	169.0143; 125.0248
5	1.19	C_10_H_14_N_2_O_5_	[M—H]^–^	241.0831	241.08328	0.5	Thymidine	▴	✓	241.0831; 150.9140
6	1.28	C_7_H_6_O_4_	[M—H]^–^	153.0198	153.0194	0.4	Protocatechuic acid	▴	✓	153.0128; 135.0085; 109.0297
7	1.42	C_8_H_8_O_4_	[M—H]^–^	167.0365	167.0354	2.2	Vanillic acid	✓	✓	167.0353
8	1.76	C_6_H_8_O_7_	[M—H]^–^	191.0204	191.0197	0.1	Citric acid	▴	✓	191.0338; 173.0223; 111.0084
9	1.92	C_9_H_8_O_4_	[M—H]^–^	179.0359	179.0352	1.3	Caffeic acid	✓	✓	179.0353; 135.0454; 117.0322
10	1.96	C_13_H_16_O_8_	[M—H]^–^	299.0775	299.0772	0.9	p-hydroxy-benzoyl-β-D-glucopyranoside	▴	✓	299.0778; 137.0248
11	2.33	C_7_H_6_O_3_	[M—H]^–^	137.0246	137.0240	1	Protocatechualdehyde	▴	✓	137.0246; 119.0124
12	3.18	C_16_H_18_O_9_	[M—H]^–^	353.0878	353.0876	-0.1	Cryptochlorogenic acid	▴	✓	353.0884; 191.0558; 135.0521
13	3.27	C_7_H_6_O_2_	[M—H]^–^	121.0291	121.0355	-3.7	4-Hydroxybenzaldehyde	▴	✓	121.0292;
14	4.05	C_13_H_16_O_7_	[M—H]^–^	283.0823	283.0826	-0.1	4-O-α-L-Rhamnopyranosylbenzoic acid	▴	✓	283.0821; 239.0888; 171.0924; 137.0236
15	4.08	C_8_H_8_O_2_	[M—H]^–^	135.0456	135.0445	1.6	4-Methylbenzoic acid	✓	✓	135.0465;
16	4.96	C_21_H_20_O_11_	[M—H]^–^	447.0926	447.0936	-1.5	Isoorientin	✓	✓	447.0908; 357.0613; 327.0502; 285.0407
17	5.22	C_26_H_28_O_14_	[M—H]^–^	563.1393	563.1416	-2.3	Schaftoside	▴	✓	563.1396; 473.1062; 443.0971; 383.0775; 353.0673
18	5.77	C_21_H_20_O_10_	[M—H]^–^	431.0979	431.0985	-1.2	Vitexin	▴	✓	431.0987; 341.0660; 311.0561; 283.0616;
19	6.07	C_22_H_22_O_10_	[M—H]^–^	445.1143	445.1140	0.7	3′-Methoxypuerarin	▴	✓	445.1112; 297.0383; 282.0549;
20	6.87	C_10_H_10_O_4_	[M—H]^–^	193.0511	193.0519	2.3	Ferulic Acid	▴	✓	193.0515; 178.0284; 134.0383;
21	7.25	C_23_H_24_O_12_	[M—H]^–^	491.1192	491.1198	-0.6	Tricin 7-O-glucoside	▴	✓	491.1206; 461.0742; 313.0341; 271.0253
22	8.28	C_27_H_26_O_11_	[M—H]^–^	525.1398	525.1412	-0.9	Salcolin B	✓	✓	525.1515; 447.1103; 329.0668; 314.0426; 299.0206; 195.0665
23	8.31	C_33_H_36_O_16_	[M—H]^–^	687.1934	687.1931	0.6	Tricin 4′-O-(erythro-beta-guaiacylglyceryl) ether 7-O-beta-D-glucopyranoside	▴	✓	687.1957; 525.1354; 491.1174; 329.0653; 195.0659
24	8.65	C_18_H_36_O_2_	[M—H]^–^	283.0607	283.0603	0.9	Wogonin	✓	✓	283.0607; 268.0381; 211.0390;
25	8.72	C_9_H_10_O_5_	[M—H]^–^	197.0458	197.0446	1.4	Syringic acid	▴	✓	197.0458; 182.0220; 167.0009; 123.0097
26	9.01	C_20_H_20_O_7_	[M—H]^–^	371.1133	371.1143	-0.8	Tangeretin	▴	✓	371.1134; 327.1244; 297.1137; 267.0634; 146.0374
27	9.29	C_21_H_22_O_8_	[M—H]^–^	401.1237	401.1243	-1.1	Nobiletin	▴	✓	401.1218; 357.1268; 311.0554; 237.0732
28	9.51	C_27_H_24_O_10_	[M—H]^–^	507.1298	507.1335	0.3	(5S,6S)-5,6-dihydro-3,8,10-trihydroxy-5-(4-hydroxy-3-methoxyphenyl)-6-hydroxymethyl-2,4-dimethoxy-7H-benzo	▴	✓	507.1304; 492.1015; 447.0859; 461.0834; 446.0645; 339.0505
29	10.18	C_16_H_14_O_6_	[M—H]^–^	301.0718	301.0722	0.1	7-O-Methyleriodictyol	▴	✓	301.0704; 286.0469; 243.0310; 215.0353
30	10.2	C_17_H_14_O_7_	[M—H]^–^	329.0664	329.0673	-1	Tricin	✓	✓	329.0663; 314.0417; 299.0193; 271.0245; 227.0341
31	10.91	C_22_H_22_O_8_	[M—H]^–^	413.1241	413.1248	-0.5	Podophyllotoxin	▴	✓	413.1236; 235.0603; 193.0502; 163.0401
32	12.25	C_13_H_24_O_4_	[M—H]^–^	243.1645	243.1602	1.2	Dodecanedioic acid monomethyl ester	▴	✓	243.1635; 225.1494; 181.1605; 130.9666
33	12.99	C_13_H_18_O_3_	[M—H]^–^	221.1185	221.1171	0.8	Dehydrovomifoliol	✓	✓	221.1187; 177.1314; 146.0953
34	16.77	C_15_H_10_O_6_	[M—H]^–^	285.2051	285.2062	-1.4	Kaempferol	✓	✓	285.2062; 267.1960; 223.2066; 148.9568
35	17.1	C_20_H_26_O_6_	[M—H]^–^	361.1631	361.1660	-2.4	(+)-Secoisolariciresinol	▴	✓	361.1567; 293.1746; 236.1052; 221.1539
36	18.83	C_27_H_30_O_16_	[M—H]^–^	609.1452	609.1446	-1.4	Kaempferol 3-gentiobioside	▴	✓	609.1458; 447.0924; 357.0637; 327.0509
37	20.72	C_20_H_24_O_3_	[M—H]^–^	311.1684	311.1650	1.8	Triptophenolide	▴	✓	311.1682; 183.0120
38	24.15	C_17_H_24_O_3_	[M—H]^–^	275.1666	275.1659	0.9	6-Shogaol	✓	✓	275.1671; 259.1195
39	25.2	C_37_H_46_O_12_	[M—H]^–^	681.2938	681.2922	3.2	Agrimol B	▴	✓	681.2946; 653.3015
40	25.32	C_16_H_32_O_2_	[M—H]^–^	255.2331	255.2330	0.2	13-methyl Myristic Acid methyl ester	▴	✓	255.2318; 214.9965
41	25.4	C_11_H_20_O_2_	[M—H]^–^	183.1394	183.1379	1.7	Methyl (E)-4-decenoate	▴	✓	183.1391; 116.9959

Rt, retention time; SDRS, SD rat serum; √ compound detected; ▴ compound not detected; PIS, phytochemical in sugarcane leaf extract.

### 3.2 Effects of oral administration of sugarcane leaf extract on rat behavior

**Figure 2:**
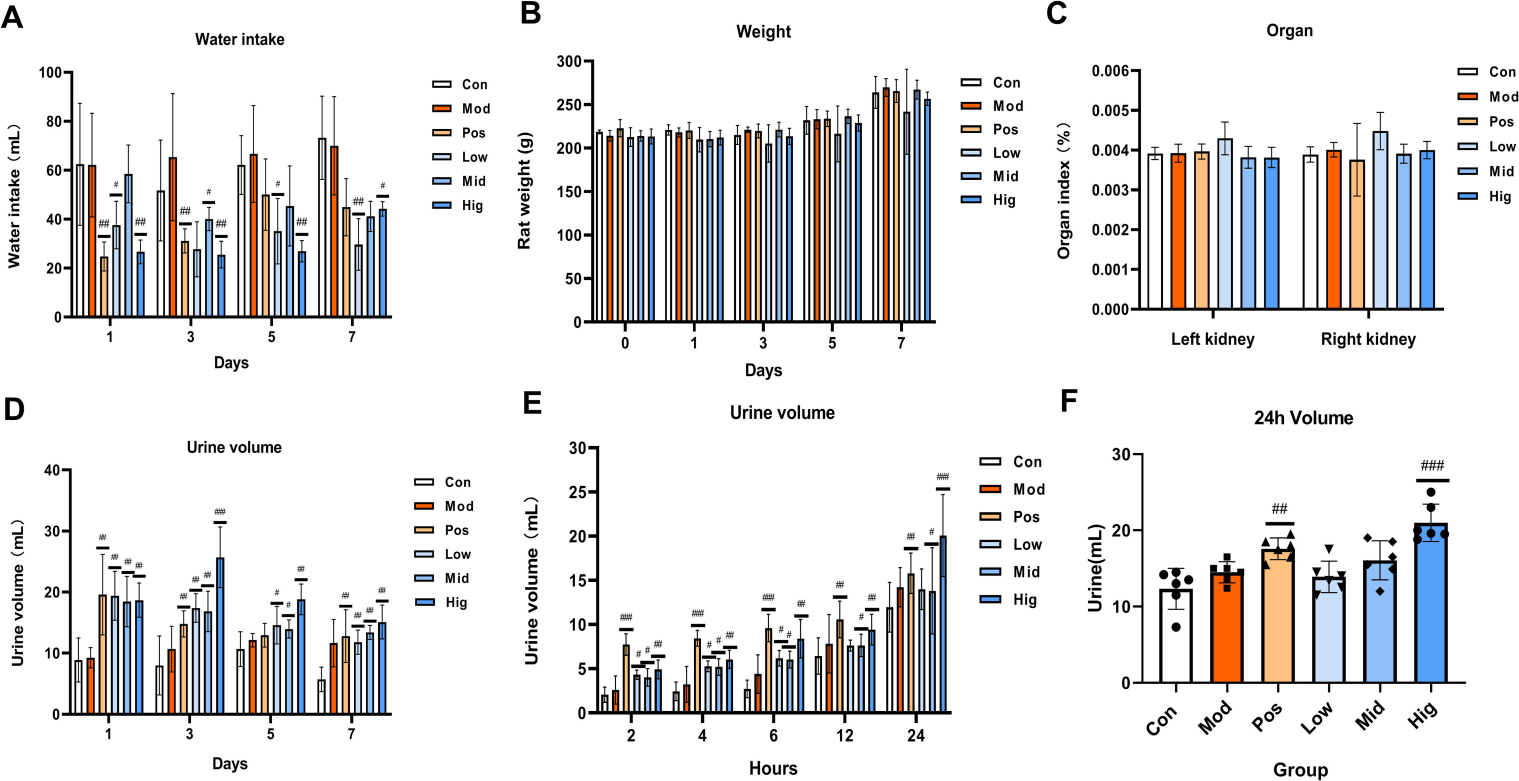
Effects of oral administration of sugarcane leaf extract on rat behavior, including water intake, rat body weight, rat kidney organ index, and urine volume at different time points. Compared with the model group, n = 6, ^#^ *P* < 0.05, ^##^ *P* < 0.01, ^###^ *P* < 0.001, ordinary One-way ANOVA. Values are expressed as mean ± SEM. The experiment was divided into six groups: blank control (Con), model (Mod), positive control (Pos), low-dose sugarcane leaf extract (Low), middle-dose sugarcane leaf extract (Mid), and high-dose sugarcane leaf extract (Hig).

During treatment, sugarcane leaf extract-administered rats displayed normal mental status without anxiety/depression behaviors. Food consumption remained stable with mild hyperphagia tendencies. Animals demonstrated frequent spontaneous movement and normal grip strength in metabolic cages. Coat condition remained normal (smooth, white) without alopecia or dermatological lesions. sugarcane leaf extract treatment significantly decreased water intake versus Control and Model groups (*P* < 0.01; **Figure 2A**). Body weight trajectories were comparable among groups, with marginal increase at day 7 (**Figure 2B**). Renal tissue weight changes are presented in **Figure 2C**.

Urine samples were collected from all groups at days 1, 3, 5, and 7. Compared to the Con group, urine volume in all groups significantly increased. At day 3, the urine volume of the Hig group exhibited maximal diuresis versus other groups (*P* < 0.01; **Figure 2D**). On day 7 of oral administration, urine samples were collected at different time points (2, 4, 6, 12, and 24 h). During 0–12 h, the urine volume of the Pos group was higher than that of the Con group, the Mod group, and the sugarcane leaf treatment groups (**Figure 2E**). However, from 12–24 h, the urine volume of the Hig group was significantly higher than that of the Mod group (*P* < 0.001) (**Figure 2F**).

### 3.3 Effects of sugarcane leaf extract on the electrolyte content in rat urine and serum

**Figure 3:**
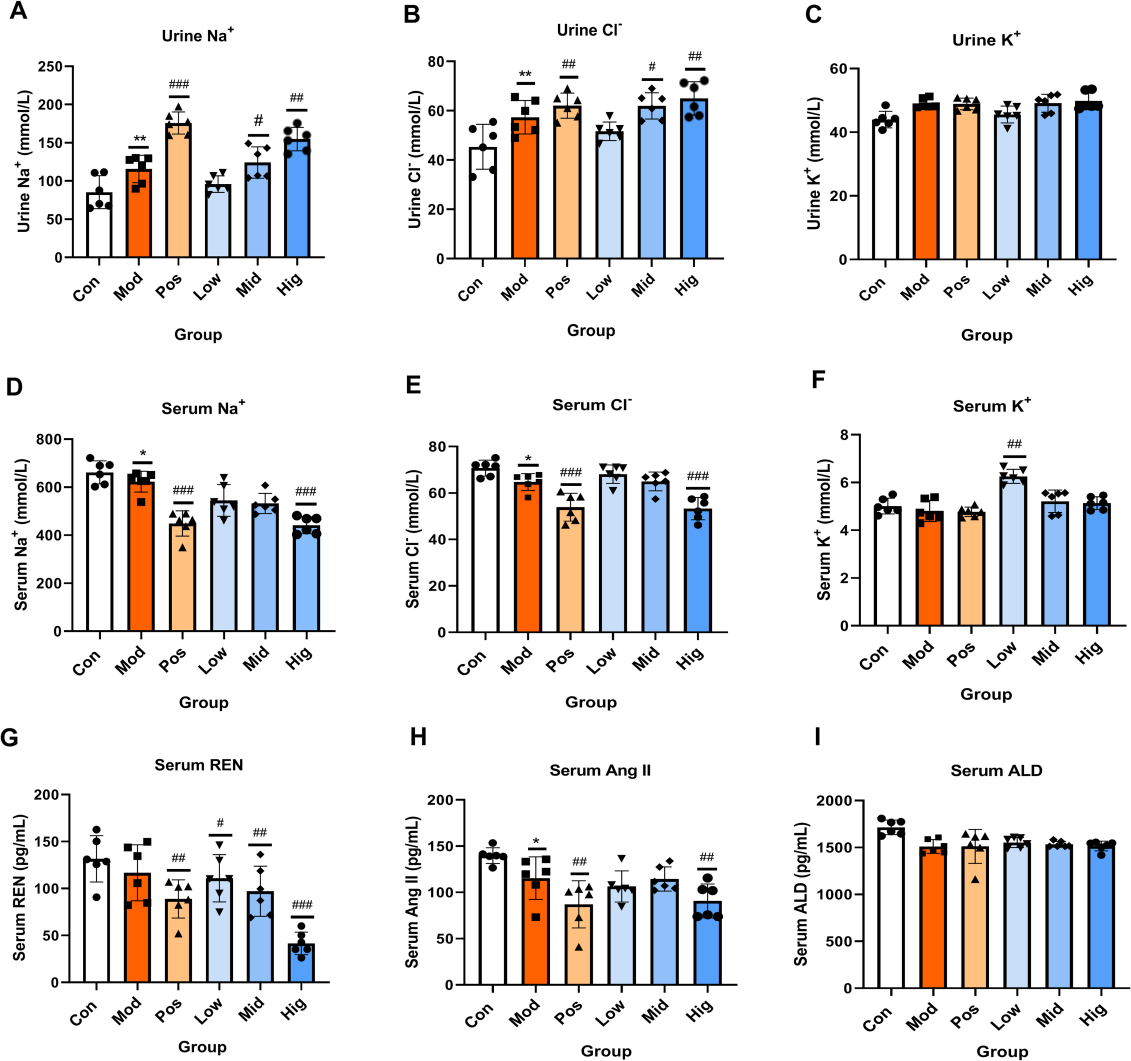
Effects of sugarcane leaf extract on the trends of Na^+^, Cl^-^, K^+^ electrolyte changes in rat urine and serum, as well as on the renin-angiotensin-aldosterone system. **(A-F)** Comparative analysis of Na^+^, Cl^-^, K^+^ in urine and serum. **(G-I)** Expression levels of REN, Ang II, and ALD in the renin-angiotensin-aldosterone system in the serum of rats from each group. Compared with the model group, n = 6, ^#^ *P* < 0.05, ^##^ *P* < 0.01, ^###^ *P* < 0.001; compared with the blank control group, ^*^ *P* < 0.05, ^**^ *P* < 0.01. Ordinary One-way ANOVA. Values are expressed as mean ± SEM. Theexperiment was divided into six groups: blank control (Con), model (Mod), positive control (Pos), low-dose sugarcane leaf extract (Low), middle-dose sugarcane leaf extract (Mid), and high-dose sugarcane leaf extract (Hig).

In rat urine, compared with the Mod group, the levels of Na^+^ and Cl^−^ ions in the Pos group, and Hig group were significantly increased (*P* < 0.01), while there were no significant changes in the levels of K^+^ ions among all groups. In rat serum, compared with the Mod group, the levels of Na^+^ and Cl^−^ ions in the Pos group and Hig group were significantly decreased (*P* < 0.001). In contrast, the levels of K^+^ ions were significantly increased in the Low group. These findings demonstrate sugarcane leaf extract-mediated promotion of the Na^+^ and Cl^−^ excretion, resulting in significant diuresis. Additionally, REN, Ang II, and ALD, in the renin-angiotensin-aldosterone system (RAAS) were quantified in serum. Compared with the Con group, the levels of REN and Ang II in the Hig group and Pos group were significantly decreased in a concentration-dependent manner (*P* < 0.01). ALD levels showed nonsignificant decreasing trends across treatment groups. Data are presented in **Figure 3**.

**Figure 4:**
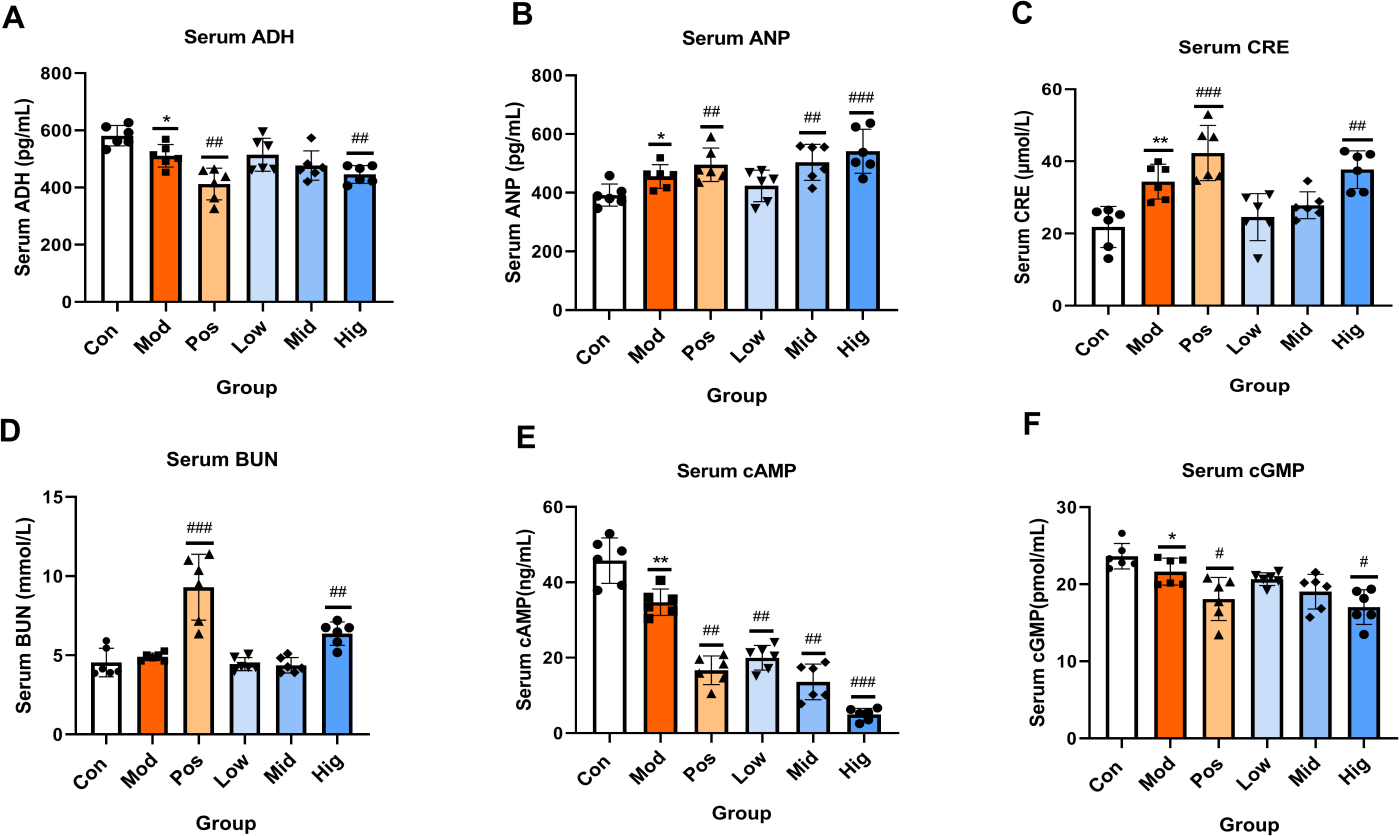
Effects of sugarcane leaf extract on antidiuretic hormone (ADH), atrial natriuretic peptide (ANP), creatinine (CRE), blood urea nitrogen (BUN), cyclic adenosine monophosphate (cAMP), and cyclic guanosine monophosphate (cGMP) in rat serum. Compared with the model group, n = 6, ^#^ *P* < 0.05, ^##^ *P* < 0.01, ^###^ *P* < 0.001; compared with the blank control group, ^*^ *P* < 0.05, ^**^ *P* < 0.01. Ordinary One-way ANOVA. Values are expressed as mean ± SEM. Theexperiment was divided into six groups: blank control (Con), model (Mod), positive control (Pos), low-dose sugarcane leaf extract (Low), middle-dose sugarcane leaf extract (Mid), and high-dose sugarcane leaf extract (Hig).

Serum levels of ADH, ANP, CRE, BUN, cAMP, and cGMP were quantified. The results showed that compared with the Con group, the levels of ADH in the Hig group and Pos group were significantly reduced. Compared with the Con group, the levels of ANP in the Hig group, Mid group, and Pos group were significantly increased (*P* < 0.01). Compared with the Con group, the levels of CRE and BUN of the Hig group were significantly elevated (*P* < 0.01). Compared with the Con group, the levels of cAMP in the sugarcane leaf treatment groups were significantly decreased (*P* < 0.01) in a dose-dependent manner. Compared with the Con group, the level of cGMP in the Hig group was reduced (*P* < 0.05). Data are presented in **Figure 4**.

### 3.4 Evaluation of the effects of sugarcane leaf extract on renal histopathology in rats

**Figure 5:**
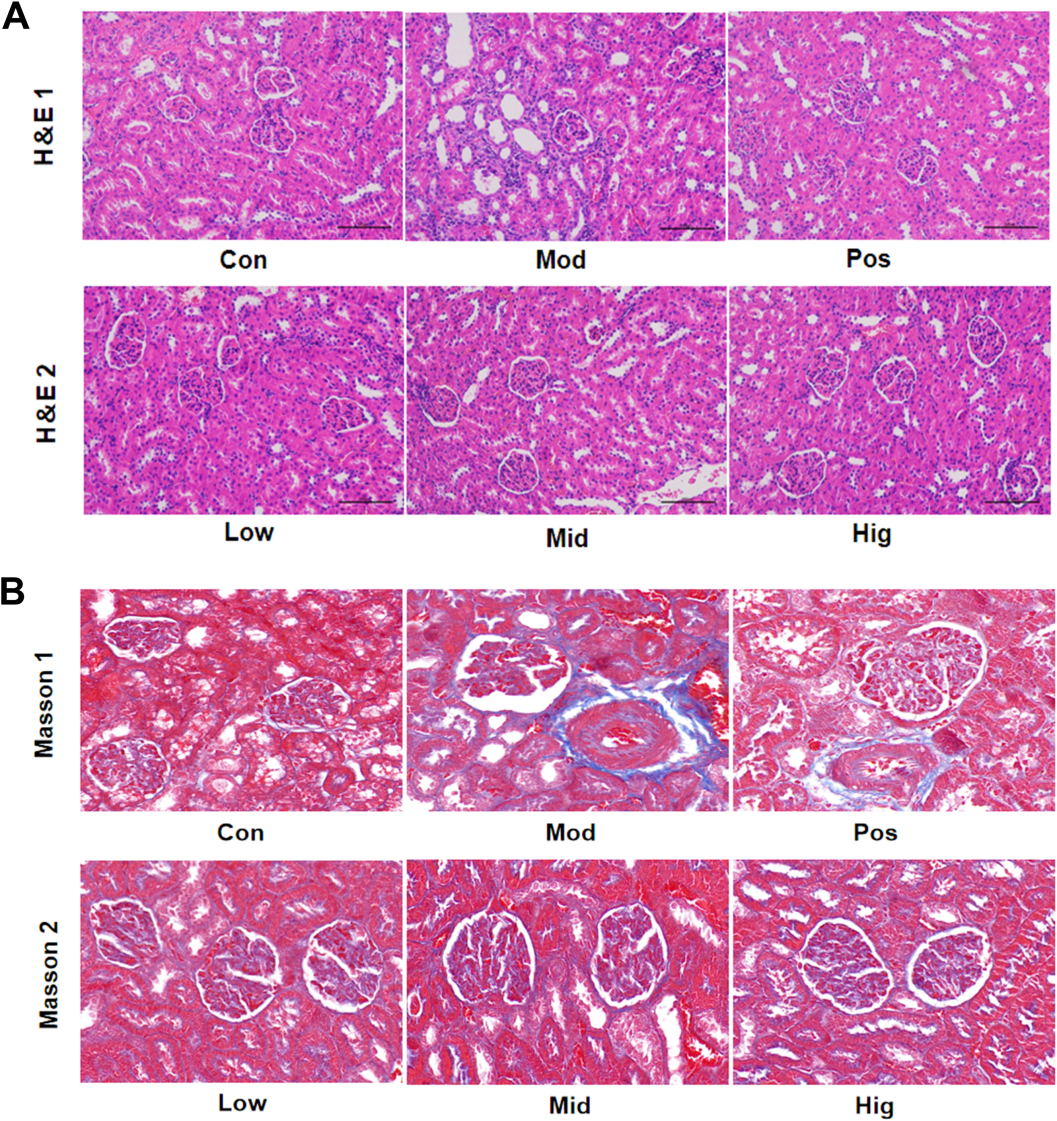
Effects of sugarcane leaf extract on rat kidney tissue pathology. **(A)** After H&E staining of the renal cortex in rats, compared with the Mod group, the glomerular structures of the other sugarcane leaf treatment groups were clear, with no significant differences in morphology. **(B)** After Masson staining of the renal cortex, compared with the Mod group, there was no obvious collagen fiber proliferation in the glomeruli and no significant collagen fiber distribution in the interstitial areas around the renal tubules in the sugarcane leaf treatment groups.

H&E staining (**Figure 5A**) demonstrated glomerular swelling, structural irregularities, and mild leukocyte infiltration in Mod group renal cortex. In contrast, the sugarcane leaf treatment groups displayed clear glomerular structures without significant morphological differences. The mesangial cells and extracellular matrix were normally distributed, with no abnormal dilation in the capillary lumina or Bowman’s space. Additionally, renal tubular cells and interstitial tissue showed no obvious pathological changes. Furthermore, Masson staining of the renal cortex (**Figure 5B**) demonstrated that the Mod group had significantly increased collagen fibrosis, altered glomerular morphology, and enhanced collagen deposition. Peritubular collagen fibers were observed around renal tubular epithelial cells, indicating kidney injury in the Mod group. In contrast, the sugarcane leaf treatment groups exhibited no obvious glomerular collagen hyperplasia, and minimal collagen fiber distribution was detected in the peritubular interstitium, suggesting that sugarcane leaf extract did not induce significant renal damage.

### 3.5 Screening of endogenous differential metabolic biomarkers and metabolic pathway analysis

**Figure 6:**
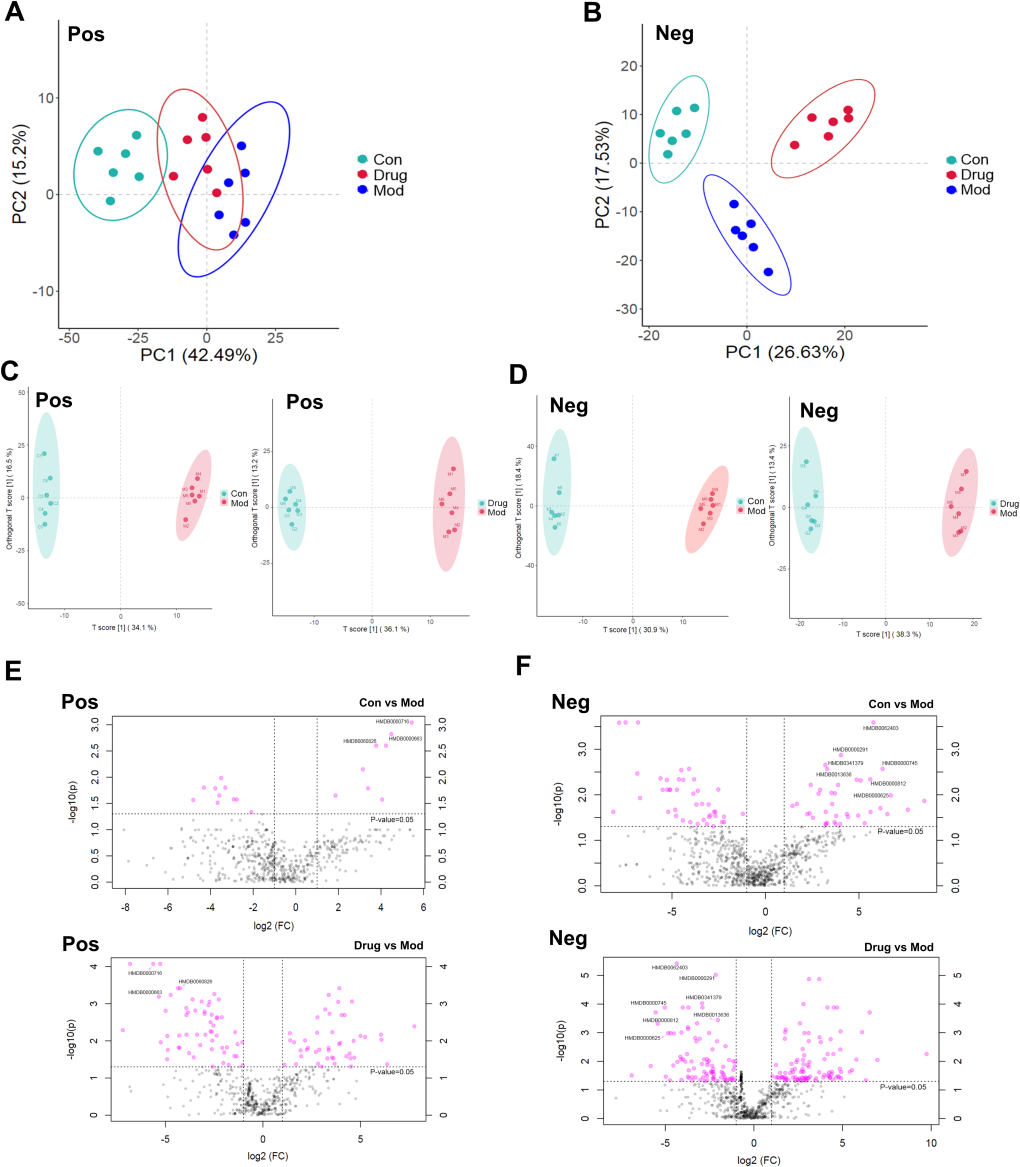
Scores of PCA, OPLS–DA, and differential volcano plots of rat urine samples. **(A)** PCA analysis in positive ion mode. **(B)** PCA analysis in negative ion mode. **(C)** OPLS–DA analysis in positive ion mode. **(D)** OPLS–DA analysis in negative ion mode. **(E)** Distribution of differential metabolites in positive ion mode. **(F)** Distribution of differential metabolites in negative ion mode.

The PCA of urinary metabolomes demonstrated clear separation between Con and Mod groups in both positive and negative ion modes. Sugarcane leaf extract treatment induced metabolic profile shifts from Model towards Control clusters, indicating metabolic normalization (**Figures 6A, B**). OPLS-DA analysis (positive mode) showed Control vs Model variance contributions of 34.1% (t-score) and 16.5% (orthogonal t-score). Negative ion mode values were 30.9% and 18.4%, respectively. Model vs sugarcane leaf extract comparisons yielded 36.1%/13.2% (positive) and 38.3%/13.4% (negative mode) variance explanations. All samples clustered within 95% confidence ellipses with distinct separation, confirming model robustness (**Figures 6C, D**).

**Figure 7:**
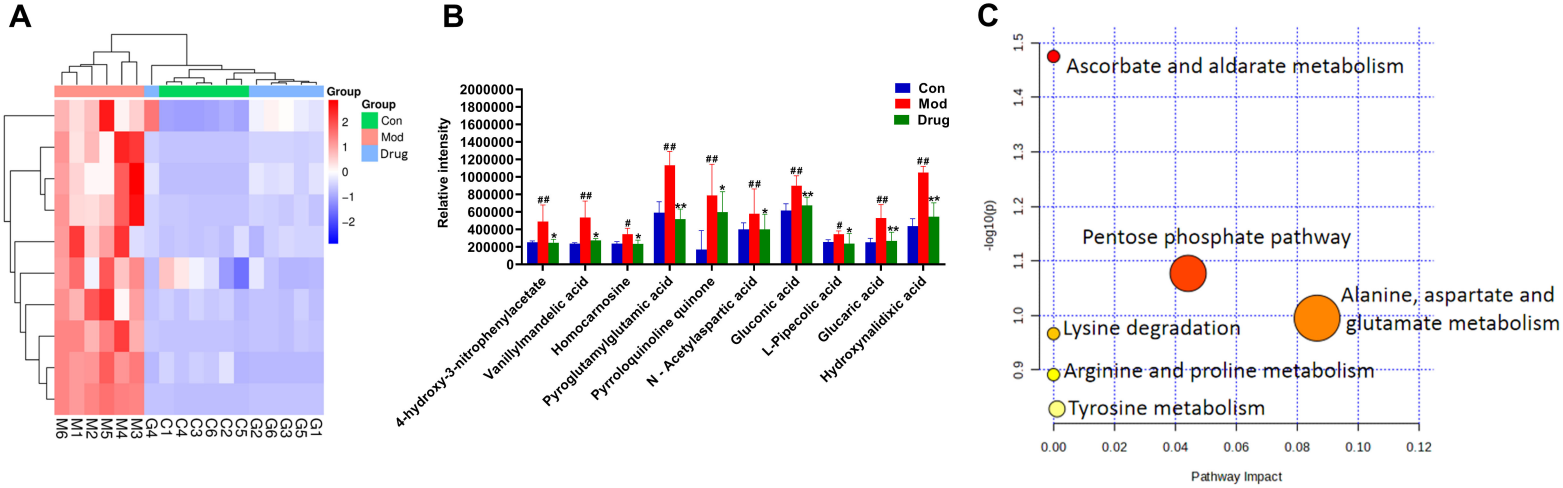
Expression levels of endogenous differential metabolic biomarkers and enrichment analysis of metabolic pathways. **(A)** Heatmap showing the expression levels of differential metabolites in different groups. **(B)** Bar chart representing the changes in the relative intensity of differential metabolites in different groups. **(C)** Pathway enrichment analysis of differential metabolites. Compared with the blank control group, ^#^ *P* < 0.05, ^##^ *P* < 0.01; compared with the model group, * *P* < 0.05, ** *P* < 0.01, mean ± SEM.

Significant metabolites were identified using combined thresholds (*P* < 0.05 from Student’s t-test and VIP > 1 from OPLS-DA analysis). Volcano plots (**Figures 6E, F**) highlight significantly altered metabolites (pink) among Control, Model and sugarcane leaf extract groups. Ten endogenous biomarkers were identified across both ionization modes, with corresponding heatmaps and intensity profiles (**Figures 7A, B**). Pathway enrichment analysis associated these biomarkers with: alanine-aspartate-glutamate metabolism, pentose phosphate pathway, ascorbate/aldarate metabolism, and tyrosine metabolism (**Figure 7C**). Biomarker characteristics are detailed in **Table 2**.

**Table 2:** Identification of endogenous differential metabolic biomarkers based on untargeted metabolomics.

No.	Metabolite name	HMDB ID	Molecular formula	Retention time	Adduction	Molecular weight	Con vs mod	Mod vs drug
1	4-hydroxy-3-nitrophenylacetate	HMDB0062403	C_8_H_7_NO_5_	6.42	[M—H]^–^	196.0977	up	down
2	Vanillylmandelic acid	HMDB0000291	C_9_H_10_O_5_	6.52	[M—H]^–^	197.1176	up	down
3	Homocarnosine	HMDB0000745	C_10_H_16_N_4_O_3_	4.61	[M—H]^–^	239.0933	up	down
4	Pyroglutamylglutamic acid	HMDB0341379	C_10_H_14_N_2_O_6_	4.30	[M—H]^–^	257.1027	up	down
5	Pyrroloquinoline quinone	HMDB0013636	C_14_H_6_N_2_O_8_	2.96	[M—H]^–^	329.0464	up	down
6	N-Acetylaspartic acid	HMDB0000812	C_6_H_9_NO_5_	7.78	[M—H]^–^	174.9565	up	down
7	Gluconic acid	HMDB0000625	C_6_H_12_O_7_	4.48	[M—H]^–^	195.0663	up	down
8	L-Pipecolic acid	HMDB0000716	C_6_H_11_NO_2_	7.40	[M+H]^+^	130.0644	up	down
9	Glucaric acid	HMDB0000663	C_6_H_10_O_8_	5.51	[M+H]^+^	211.1334	up	down
10	Hydroxynalidixic acid	HMDB0060826	C_12_H_12_N_2_O_4_	9.53	[M+H]^+^	249.1849	up	down

### 3.6 Network pharmacology analysis to screen key targets for the diuretic effect of sugarcane leaf extract

**Figure 8:**
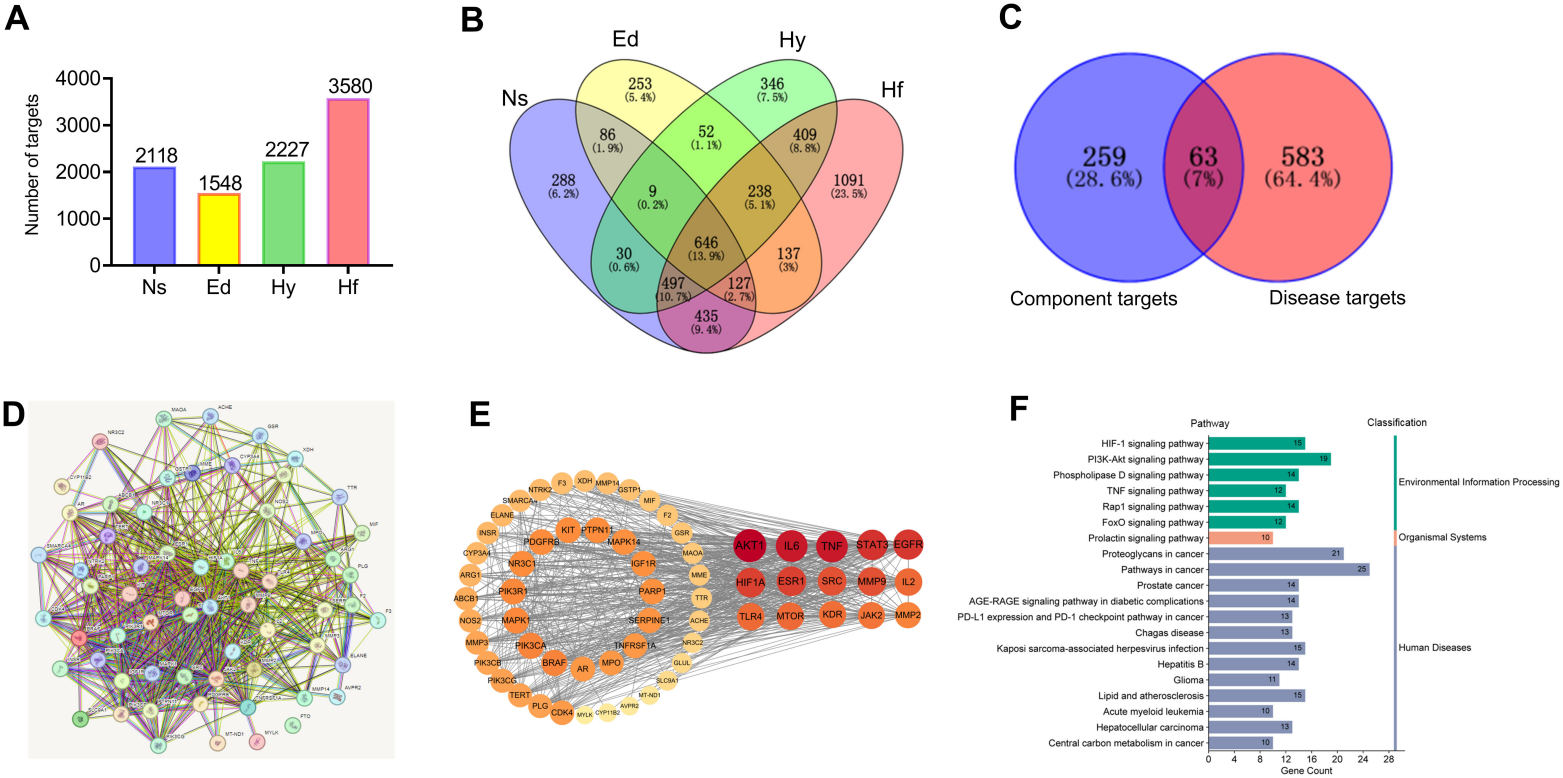
Network pharmacology analysis of the diuretic effect of sugarcane leaf extract. **(A)** Disease targets obtained using the GenCards, OMIM, and DisGeNET databases. **(B)** Intersection of disease targets obtained using a Venn diagram. **(C)** Identification of 63 potential targets for the diuretic effect of sugarcane leaf extract from the overlap of “component targets - disease targets” in the Venn diagram. **(D)** PPI network of potential targets. **(E)** Top 15 key targets obtained by calculating with the Centiscape 2.2 plugin tool in Cytoscape 3.10.0 software. **(F)** KEGG pathway enrichment classification of potential targets.

Ten serum-absorbed components identified via UPLC–MS were considered potential sugarcane leaf extract bioactive constituents, likely constituting the pharmacological basis for its diuresis. These 10 blood components of sugarcane leaf extract, 322 drug targets were predicted from the Swiss Target Prediction database and the SEA database. Using the GeneCards, OMIM, and DisGeNET disease databases, 1,548 targets for edema, 2,227 targets for hypertension, 3,580 targets for heart failure, and 2,118 targets for nephrotic syndrome were obtained. Target intersection yielded 646 unique disease targets. From the Venn diagram of the overlap between “component targets and disease targets”, 63 targets were identified as potential targets for the diuretic effect of sugarcane leaf extract (**Figures 8A–C**).

**Figure 9:**
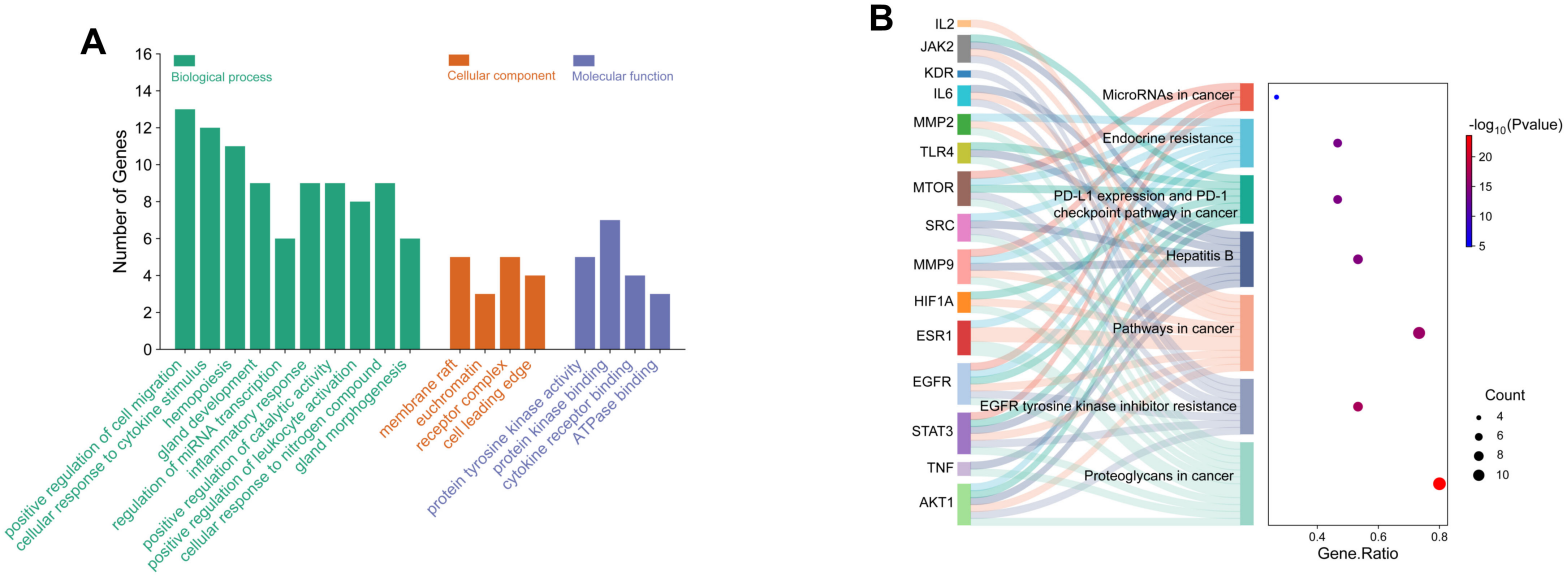
GO and KEGG pathway enrichment analysis of key targets. **(A)** GO analysis of biological processes, cellular components, and molecular functions of key targets. **(B)** KEGG pathway enrichment analysis of key targets using the enrichment Sankey diagram.

Potential targets were analyzed via STRING to build a protein-protein interaction (PPI) network (**Figure 8D**). Centiscape 2.2 plugin in Cytoscape 3.10.0 calculated network topology parameters, identifying top 15 targets by degree/betweenness/closeness centrality. This PPI network revealed key diuretic targets (**Figure 8E**). Additionally, KEGG pathway enrichment analysis was performed for all 63 targets (**Figure 8F**). Top 15 targets underwent GO enrichment analysis (**Figure 9A**). The biological processes (BP) mainly involved positive regulation of cell migration, cellular response to cytokine stimulus, and inflammatory regulation. The cellular components (CC) primarily were enriched for membrane rafts, euchromatin, and receptor complexes. Molecular functions (MF) featured protein tyrosine kinase activity, kinase binding, and cytokine receptor interactions. Sankey dot pathway enrichment displayed the KEGG pathway enrichment, which included Proteoglycans in cancer, EGFR tyrosine kinase inhibitor resistance, Pathways in cancer, Hepatitis B, PD-L1 expression and PD-1 checkpoint pathway in cancer, Endocrine resistance, and MicroRNAs in cancer. In summary, the core targets were calculated by Centiscape 2.2, and the highly connected central nodes were AKT1, IL-6, TNF, STAT-3, and EGFR, indicating that these are key targets (**Figure 9B**). The PDB IDs corresponding to these five key targets are as follows: AKT1 (7NH5), IL-6 (1ALU), TNF (5M2I), STAT-3 (6NJS), and EGFR (5HG5).

### 3.7 Molecular docking of blood components with key targets

**Figure 10:**
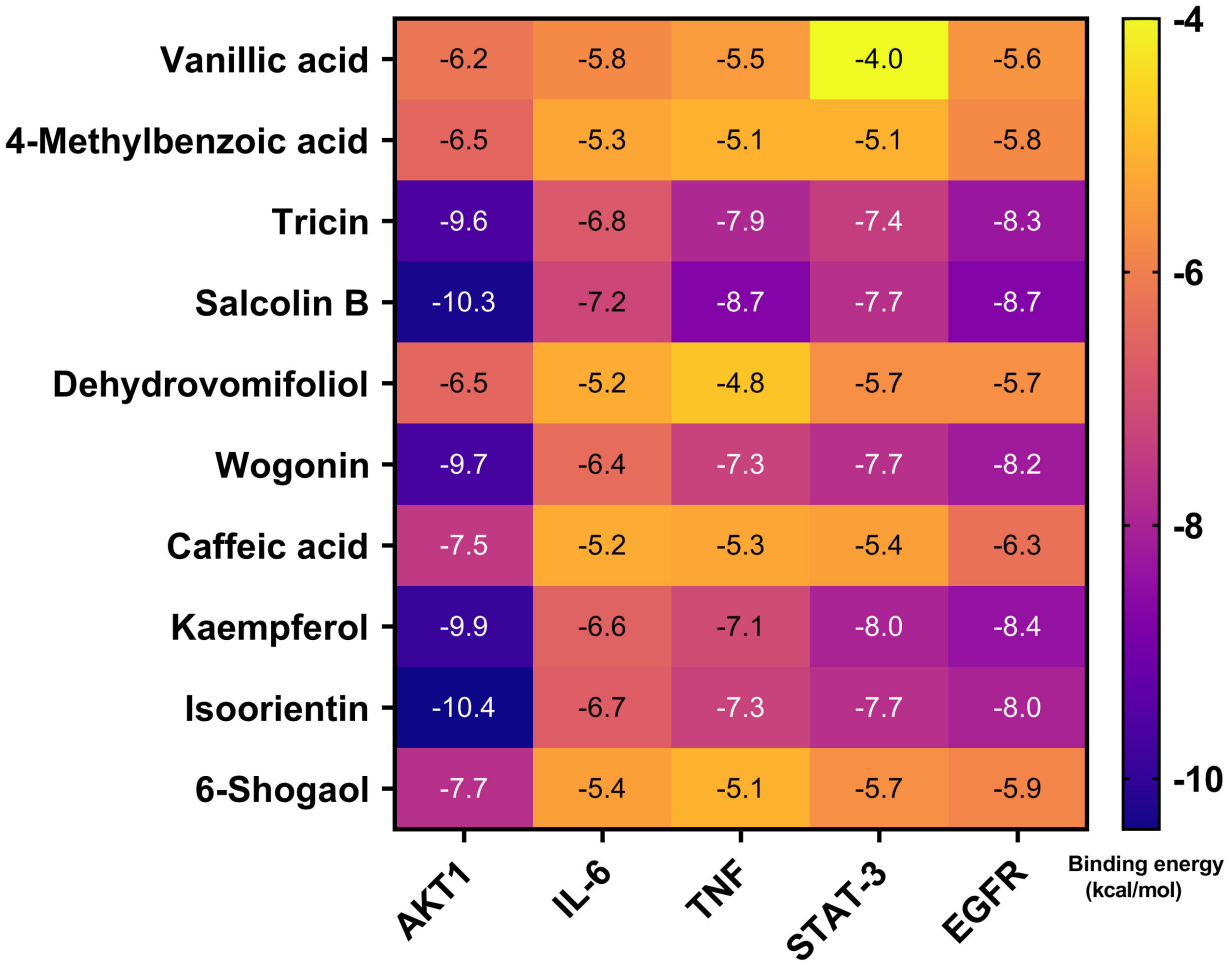
Molecular docking of the 10 blood components in sugarcane leaf extract with 5 key targets, with binding energies represented by a heatmap. The color intensity indicates the magnitude of the absolute value of the binding energy; the darker the color, the greater the absolute value of the binding energy, and the stronger the binding interaction.

**Figure 11:**
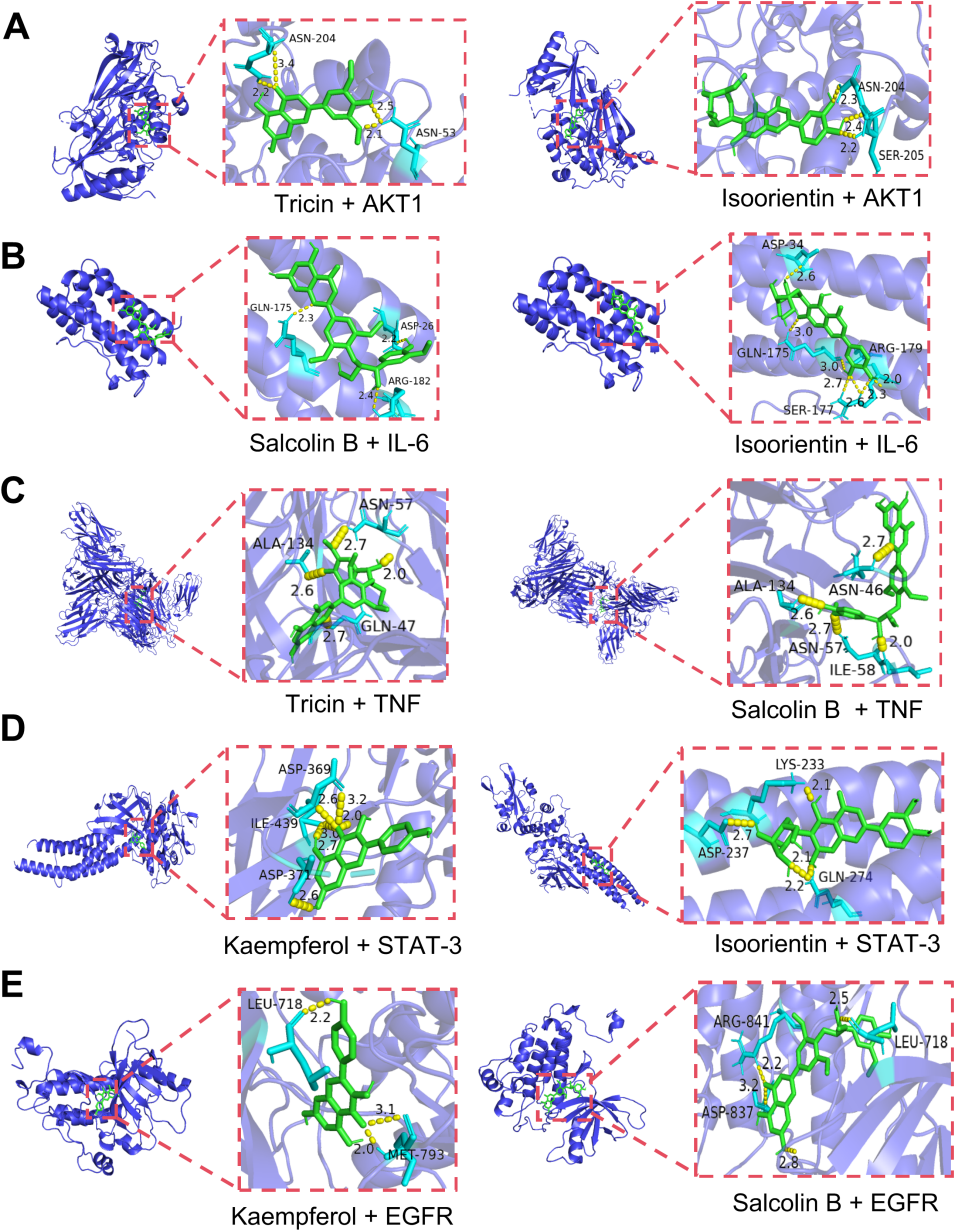
Visualization of molecular docking combinations with strong binding interactions. **(A)** Molecular docking of tricin, isoorientin with AKT1. **(B)** Molecular docking of salcolin B, isoorientin with IL-6. **(C)** Molecular docking of tricin, salcolin B with TNF. **(D)** Molecular docking of kaempferol, isoorientin with STAT-3. **(E)** Molecular docking of kaempferol, salcolin B with EGFR.

Molecular docking of 10 serum components with five core targets was performed (binding energy heatmap in **Figure 10**). The color intensity represents the magnitude of the absolute value of the binding energy, the darker the color, the greater the absolute value of the binding energy, and the stronger the binding interaction. Top binding conformations were visualized. The results showed that the binding energy of isoorientin with AKT1 was -10.4 kcal/mol, forming 2 hydrogen bonds with the SER-205 residue of AKT1 and 1 hydrogen bond with the ASN-204 residue. The binding energy of tricin with AKT1 was -9.6 kcal/mol, forming 2 hydrogen bonds with the ASN-204 residue and 2 hydrogen bonds with the ASN-53 residue of AKT1 (**Figure 11A**). Salcolin B-IL-6 binding (-7.2 kcal/mol) involved hydrogen bonds with GLN175, ASP26 and ARG182. The binding energy of isoorientin with IL-6 was -6.7 kcal/mol, forming 3 hydrogen bonds with the SER-177 residue, 1 hydrogen bond with the ASP-34 residue, and 1 hydrogen bond with the GLN-175 residue of IL-6 (**Figure 11B**). Tricin-TNF interaction (-7.9 kcal/mol) formed hydrogen bonds with ALA-134, ASN-57 and GLN-47. The binding energy of salcolin B with TNF was -8.7 kcal/mol, forming 2 hydrogen bonds with the ASN46 residue, 2 hydrogen bonds with the ALA-134 residue, and 1 hydrogen bond with the ILE-58 residue of TNF (**Figure 11C**). Kaempferol-STAT3 complex (-8.0 kcal/mol) formed three hydrogen bonds with ASN369 and one with ASP-371. The binding energy of isoorientin with STAT-3 was -7.7 kcal/mol, forming 2 hydrogen bonds with the GLN-274 residue, 1 hydrogen bond with the LYS-233 residue, and 1 hydrogen bond with the ASP-237 residue of STAT-3 (**Figure 11D**). Kaempferol-EGFR binding (-8.4 kcal/mol) involved two hydrogen bonds with MET-793 and one with LEU-718. The binding energy of Salcolin B with EGFR was -8.7 kcal/mol, forming 2 hydrogen bonds with the ARG-841 residue and 1 hydrogen bond with each of the LEG-718 and ASP-837 residues of EGFR (**Figure 11E**).

### 3.8 Molecular dynamics simulation of blood components with key targets

**Figure 12:**
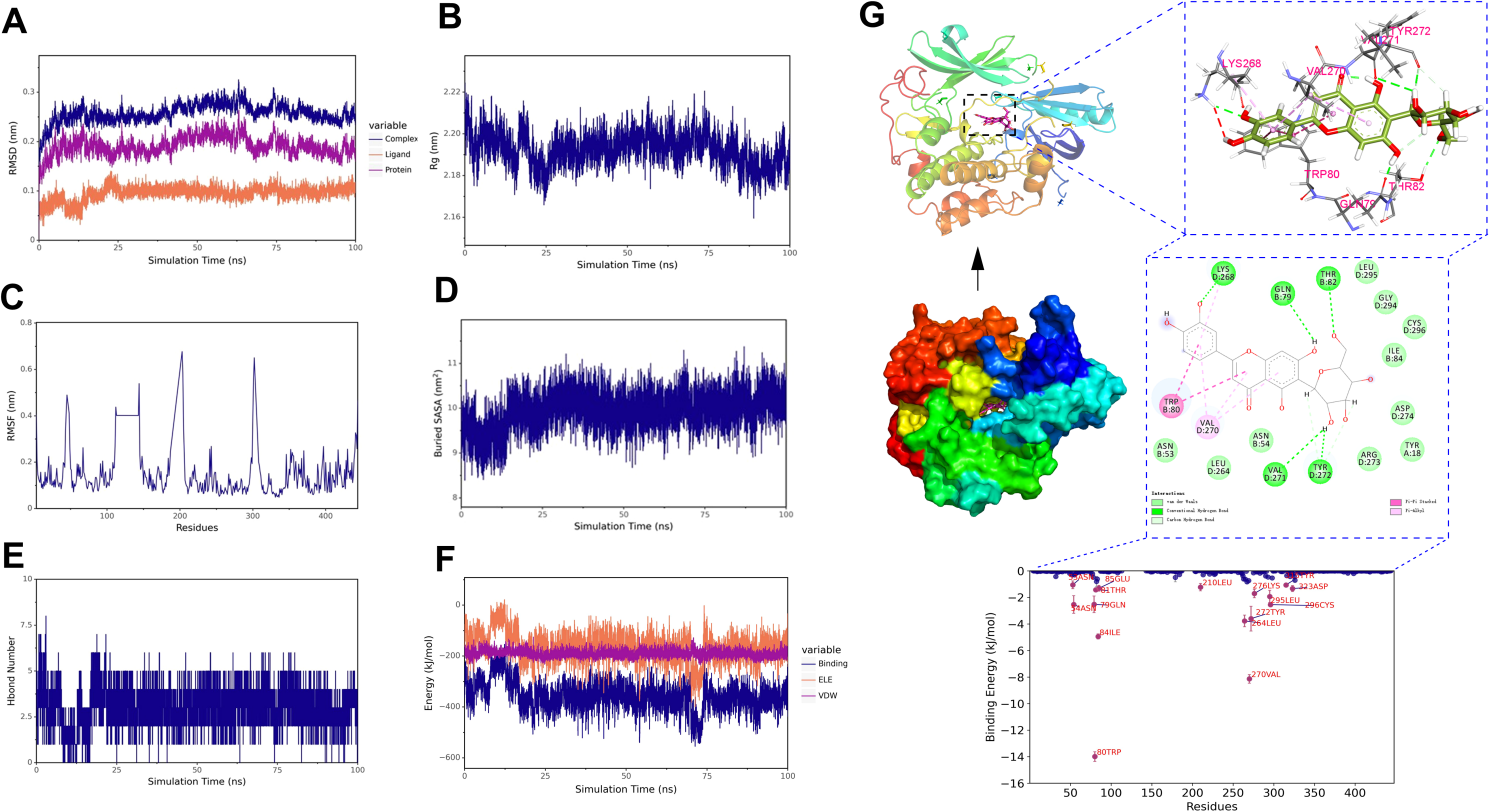
Molecular dynamics simulation of the isoorientin-AKT1 complex. **(A)** RMSD of the complex, protein, and small molecule ligand. **(B)** Rg of the complex. **(C)** RMSF of the protein in the complex. **(D)** Buried SASA between the small molecule and the protein. **(E)** Number of hydrogen bonds (Hbond number). **(F)** Binding energy (VDW and ELE) between the small molecule and the protein. **(G)** Structural and interaction analysis of isoorientin and AKT1.

To further demonstrate the degree and stability of the binding between ligands and proteins, based on the results of molecular docking, we selected two complexes for stability analysis, namely isoorientin-AKT1 and salcolin B-IL-6. For the stability and conformational analysis of isoorientin-AKT1, we calculated the RMSD, Rg, RMSF, buried SASA, hydrogen bonds, and binding energy during the molecular dynamics simulation process. The results showed that in the first half of the simulation, the RMSD deviated within the range of 0.2–0.3 nm but stabilized within 0.3 nm after 10 ns, indicating that the structure of the isoorientin-AKT1 complex remained essentially stable (**Figure 12A**). The Rg curve of the complex remained stable throughout the simulation, maintaining within the range of 2.18–2.20 nm, which indicates the stability of the complex structure (**Figure 12B**). The RMSF analysis revealed that the amino acid residues of AKT1 exhibited significant flexibility in the regions of 50–60, 110–130, 190–200, and 300–310 (**Figure 12C**). Buried SASA stabilized after 25 ns, indicating consistent binding interface (**Figure 12D**). Isoorientin maintained 2–5 stable hydrogen bonds with AKT1 (**Figure 12E**). In addition, without considering solvation, we calculated the van der Waals forces and electrostatic interactions between isoorientin and AKT1, and analyzed the changes in the binding energy of the complex during the simulation. The results showed that after 25 ns, the van der Waals forces (VDW) and electrostatic interactions (ELE) gradually stabilized with the simulation, and the binding energy of the complex also stabilized (**Figure 12F**). Structural analysis (**Figure 12B**) identified hydrogen bonds between isoorientin and AKT1 residues LYS268, GLN79, THR82, TYR272, VAL271. TRP-80, VAL-270, and LYS-268 formed π-π stacked and π-Alkyl hydrophobic interactions with the small molecule, while amino acids such as LEU-295, ASN-53, and TYR-18 formed van der Waals interactions with the small molecule (**Figure 12G**).

**Figure 13:**
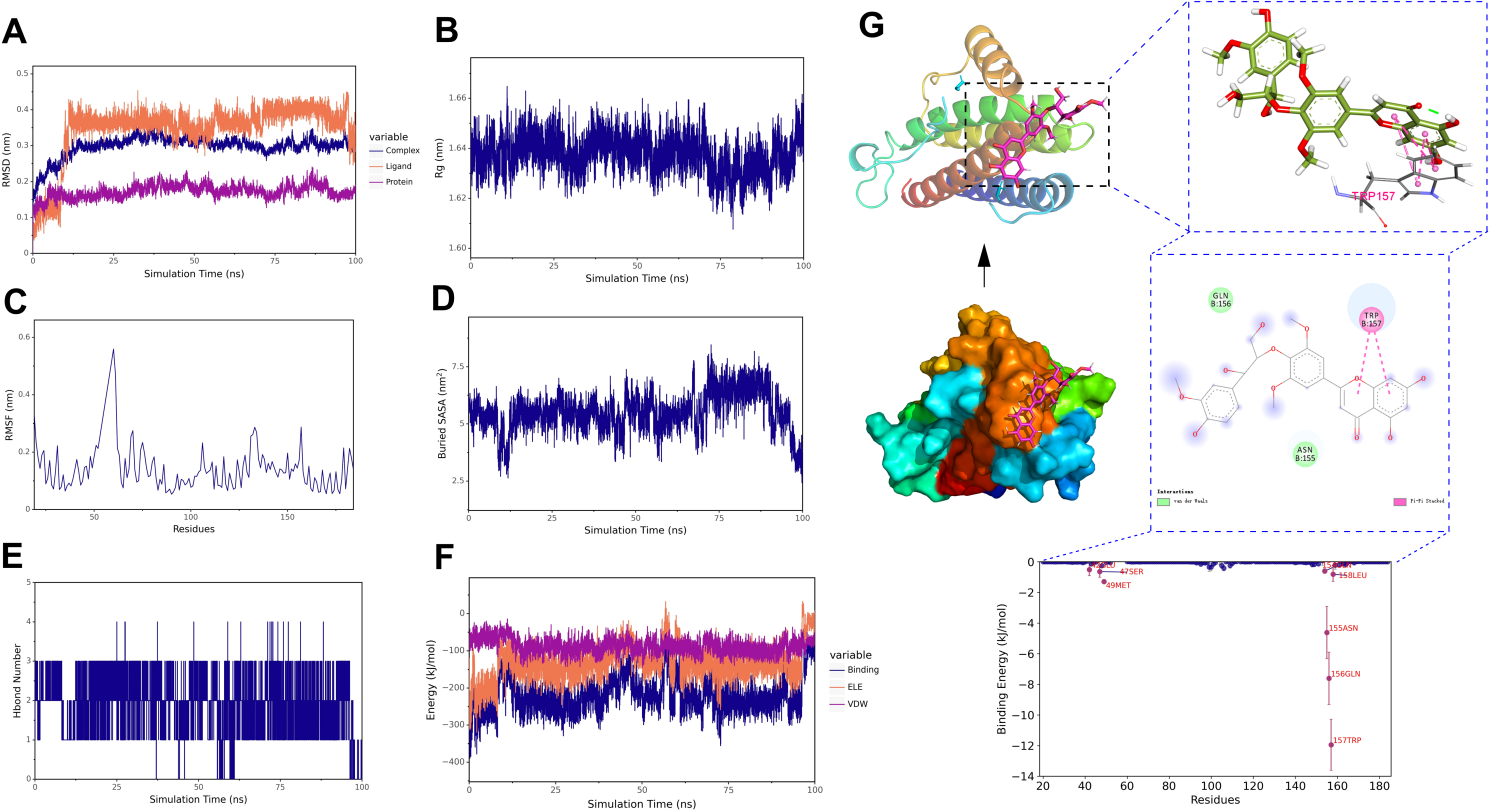
Molecular dynamics simulation of the salcolin B-IL-6 complex. **(A)** RMSD of the complex, protein, and small molecule ligand. **(B)** Rg of the complex. **(C)** RMSF of the protein in the complex. **(D)** Buried SASA between the small molecule and the protein. **(E)** Number of hydrogen bonds (Hbond number). **(F)** Binding energy (VDW and ELE) between the small molecule and the protein. **(G)** Structural and interaction analysis of salcolin B and IL-6.

For the stability and conformational analysis of the salcolin B-IL6 complex, the results showed that the RMSD deviated within the range of 0.2−0.3 nm but stabilized within 0.3 nm after 15 ns, indicating that the structure of the salcolin B-IL-6 complex remained essentially stable (**Figure 13A**). The Rg curve of the complex remained stable throughout the simulation, maintaining within the range of 1.62–1.66 nm, which indicates the stability of the complex structure (**Figure 13B**). The RMSF analysis revealed that the amino acid residues of IL-6 exhibited significant flexibility in the region of 60–70 (**Figure 13C**). The Buried SASA analysis showed that the contact area of the salcolin B-IL6 complex gradually stabilized after 15 ns (**Figure 13D**). Hydrogen bond analysis indicated that Salcolin B formed at least 1–3 hydrogen bonds with IL-6 (**Figure 13E**). In addition, without considering solvation, we calculated the van der Waals forces and electrostatic interactions between salcolin B and IL-6, and analyzed the changes in the binding energy of the complex during the simulation. The results showed that after 15 ns, the VDW and ELE gradually stabilized with the simulation, and the binding energy of the complex also stabilized (**Figure 13F**). Finally, the structural and interaction analysis of salcolin B and IL-6 revealed that TRP-157 formed a Pi-Pi stacked hydrophobic interaction with the small molecule, while amino acids such as GLN-156 and ASN-155 formed van der Waals interactions with the small molecule (**Figure 13G**).

### 3.9 Correlation analysis of endogenous biomarkers and key targets

**Figure 14:**
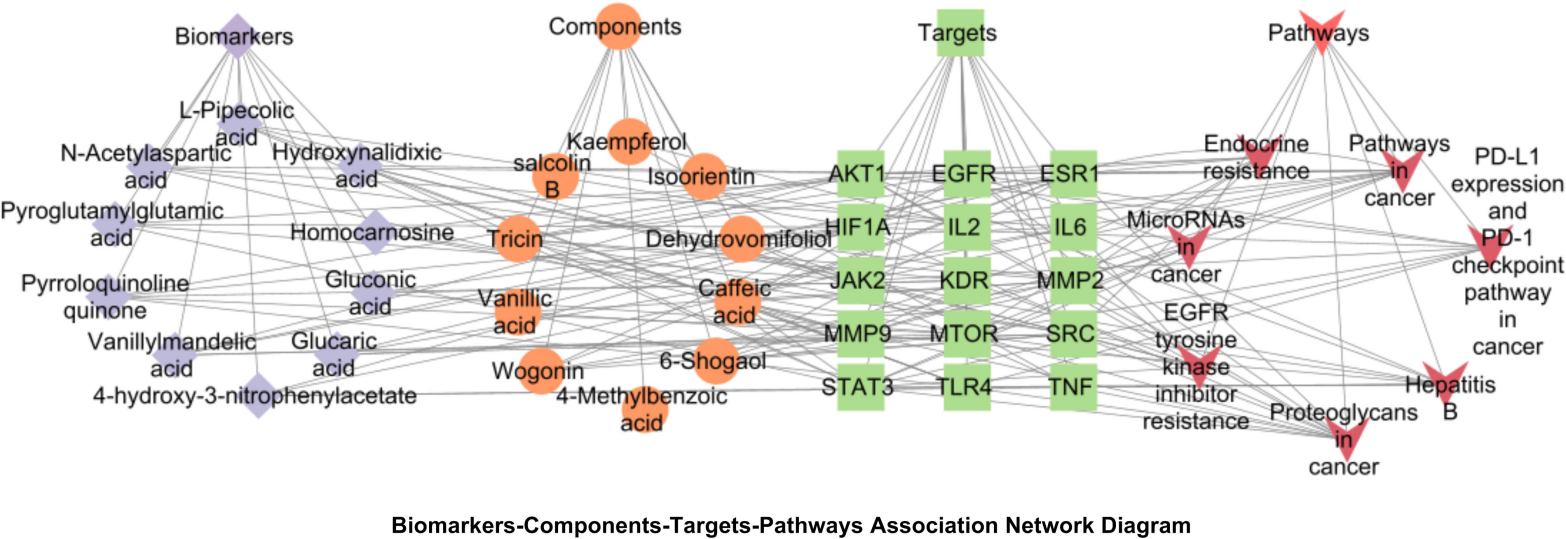
Biomarkers-components-targets-pathways network diagram of the diuretic effect of sugarcane leaf extract. Blue diamonds represent biomarkers, orange circles represent components, green squares represent key targets, and red triangles represent pathways.

Integration of untargeted metabolomics biomarkers with network pharmacology targets was performed. Cytoscape-generated network visualized biomarker-component-target-pathway relationships (**Figure 14**). The network comprised 10 biomarkers, 10 blood components, 15 core targets and 7 pathways. Three pathways were prioritized for sugarcane leaf extract’s diuretic mechanism: alanine, aspartate, and glutamate metabolism, pentose phosphate pathway, and cancer pathways.

## 4 Discussion

Serum pharmacochemistry of TCM elucidates therapeutic material basis through analyzing blood-absorbed constituents and their metabolic dynamics post-administration. These serum components act as direct bioactive mediators, interacting with biological targets and modulating pathological networks (Deng et al., 2025). In this study, UPLC–MS technology was employed to identify 10 prototype components absorbed into blood from rat serum. Among these, tricin, salcolin B, wogonin, kaempferol, and isoorientin were identified as the pharmacodynamic material basis responsible for the diuretic effects of sugarcane leaf extract.

Untargeted metabolomics is a systematic analytical approach based on high-throughput mass spectrometry that compares metabolic profile differences under various physiological/pathological states, widely employed to elucidate regulatory mechanisms of metabolic networks in organisms (Alvarez and Naldrett, 2021). This study revealed that the sugarcane leaf extract could ameliorate metabolic disorders in model rats, causing their metabolic profiles to revert towards the blank control group. Furthermore, we identified 10 endogenous differential metabolic biomarkers. The alterations in these biomarkers may be closely associated with the effects of sugarcane leaf extract. Network pharmacology provides a systematic research framework for analyzing the synergistic mechanisms of multi-component TCM (Liu et al., 2024). In this study, Swiss Target Prediction and SEA databases were employed to predict potential targets of sugarcane leaf extract. By cross-referencing with disease target databases (GeneCards, OMIM, and DisGeNET), 63 overlapping targets were identified, with AKT1, IL-6, TNF, STAT-3, and EGFR emerging as the top five core targets. These targets are involved in various biological processes including cell migration, cellular response to cytokines, and inflammatory response. Furthermore, KEGG pathway enrichment analysis revealed that the effects of sugarcane leaf extract may be mediated through the regulation of the pentose phosphate pathway and cancer-related pathways. Despite the inclusion of “cancer” in the names of these pathways, their core functions are associated with fundamental cellular processes in the kidneys, such as cell survival, proliferation, apoptosis, and inflammatory responses. The cell survival mechanisms mediated by the AKT1 and STAT3 pathways contribute to maintaining the integrity of renal tubular epithelial cells. The inflammatory responses regulated by the IL-6 and TNF pathways influence renal injury repair, thereby modulating urine production and water-electrolyte balance. Thus, these pathways are not directly involved in cancer-related pathological mechanisms but play pivotal roles in regulating renal physiological functions. Molecular dynamics simulations further validated the binding stability between the blood-absorbed components in sugarcane leaf extract and the core targets. Through simulating the dynamic behavior of the isoorientin-AKT1 and salcolin B-IL-6 complexes, we found that these components exhibit strong binding affinity with their respective targets, and the complex structures remained stable throughout the simulation process. Interestingly, in the RMSD plot of the salcolin B-IL-6 complex, it was observed that the RMSD value of the ligand rapidly increased at around 10 ns. It is speculated that the reason might be that the initial conformation of the ligand was in a local energy minimum, and the rise in RMSD during the early stage of the simulation reflected its relaxation process toward a more stable conformation. Overall, this result indicates that the active components in sugarcane leaf aqueous extract are capable of forming stable complexes with key targets. Additionally, integrating the results from untargeted metabolomics and network pharmacology analyses, this study constructed a visualized network, which further elucidated the key pathways through which sugarcane leaf extract exerts its diuretic effects. The results demonstrated that the alanine, aspartate and glutamate metabolism pathway, pentose phosphate pathway, and cancer-related pathways were the most relevant pathways involved in sugarcane leaf extract’s diuretic action. The modulation of these pathways may be closely associated with sugarcane leaf extract’s effects on improving renal function, regulating electrolyte balance, and suppressing inflammatory responses.

This study further revealed that sugarcane leaf extract could modulate the RAAS in rat serum, significantly reducing levels of REN and Ang II, which may contribute to diuretic activity. The RAAS system plays a crucial role in regulating water-electrolyte metabolism and blood pressure, and its inhibition may facilitate increased urine output and blood pressure reduction. Moreover, sugarcane leaf extract demonstrated regulatory effects on serum levels of cAMP, cGMP, ADH, ANP, CRE and BUN. These mediators critically regulate renal function and fluid-electrolyte balance. The cAMP functions as an intracellular second messenger, primarily generated by adenylyl cyclase under the control of the hormone ADH. Upon binding of ADH to V_2_ receptors on the epithelial cells of the collecting duct, adenylyl cyclase is activated, elevating cAMP levels and subsequently activating protein kinase A. activating protein kinase A phosphorylates aquaporins, promoting their insertion into the apical membrane and thereby enhancing water reabsorption (Tresguerres et al., 2011). In short, high cAMP levels suppress urine production, whereas low cAMP levels favor diuresis. The cGMP is mainly synthesized by guanylyl cyclase and is regulated by hormones such as ANP and nitric oxide. Studies indicate that cGMP modulates glomerular filtration rate and sodium excretion via the protein kinase G pathway, thereby influencing sodium and water reabsorption in the collecting duct (Shen et al., 2016). The CRE and BUN are sensitive markers for renal function screening. CRE primarily originates from muscle metabolism and is influenced by the glomerular filtration rate, while BUN is associated with protein breakdown and the hepatic urea cycle. In this study, the Hig group exhibited significantly elevated levels of Na^+^ and Cl^-^ in the urine, along with a marked increase in urine volume, indicating that sugarcane leaf extract has a diuretic activity that promotes the excretion of water and salts. Similar to loop diuretics in clinical use, which often cause a transient increase in CRE/BUN levels, this is due to the increased urine flow rate reducing the time available for renal tubular reabsorption, thereby decreasing urine concentration capacity, rather than causing damage to the renal unit structures. In summary, the diuretic activity of sugarcane leaf extract does not cause significant damage to renal tissue and should be regarded as a functional adaptation rather than a pathological condition.

## 5 Conclusions

This study systematically investigated the diuretic activity and underlying mechanisms of sugarcane leaf extract through untargeted metabolomics, network pharmacology, and molecular dynamics simulation approaches. The results demonstrated that sugarcane leaf extract possesses significant diuretic effects, markedly increasing the excretion of Na^+^ and Cl^−^ ions in urine while exerting its pharmacological actions through modulation of multiple metabolic pathways and molecular targets. These findings not only provide scientific evidence for the clinical application of sugarcane leaf, but also offer theoretical support for their development as a novel source of diuretic agents.

## Data Availability

The original contributions presented in the study are included in the article/supplementary material. Further inquiries can be directed to the corresponding authors.
